# Identification of a nanomolar affinity α-synuclein fibril imaging probe by ultra-high throughput *in silico* screening†
†Electronic supplementary information (ESI) available: *In silico* compound screening, binding assays, chemical synthesis, photo-crosslinking and fibril analysis. See DOI: 10.1039/d0sc02159h


**DOI:** 10.1039/d0sc02159h

**Published:** 2020-09-10

**Authors:** John J. Ferrie, Zsofia Lengyel-Zhand, Bieneke Janssen, Marshall G. Lougee, Sam Giannakoulias, Chia-Ju Hsieh, Vinayak Vishnu Pagar, Chi-Chang Weng, Hong Xu, Thomas J. A. Graham, Virginia M.-Y. Lee, Robert H. Mach, E. James Petersson

**Affiliations:** a Department of Chemistry , University of Pennsylvania , 231 South 34th Street , Philadelphia , PA 19104 , USA . Email: ejpetersson@sas.upenn.edu; b Department of Radiology , Perelman School of Medicine , University of Pennsylvania , Philadelphia , Pennsylvania 19104 , USA; c Center for Neurodegenerative Disease Research , University of Pennsylvania , 3600 Spruce Street , Philadelphia , PA 19104 , USA

## Abstract

Small molecules that bind with high affinity and specificity to fibrils of the α-synuclein (αS) protein have the potential to serve as positron emission tomography (PET) imaging probes to aid in the diagnosis of Parkinson's disease and related synucleinopathies. To identify such molecules, we employed an ultra-high throughput *in silico* screening strategy using idealized pseudo-ligands termed exemplars to identify compounds for experimental binding studies. For the top hit from this screen, we used photo-crosslinking to confirm its binding site and studied the structure–activity relationship of its analogs to develop multiple molecules with nanomolar affinity for αS fibrils and moderate specificity for αS over Aβ fibrils. Lastly, we demonstrated the potential of the lead analog as an imaging probe by measuring binding to αS-enriched homogenates from mouse brain tissue using a radiolabeled analog of the identified molecule. This study demonstrates the validity of our powerful new approach to the discovery of PET probes for challenging molecular targets.

## Abstract 2

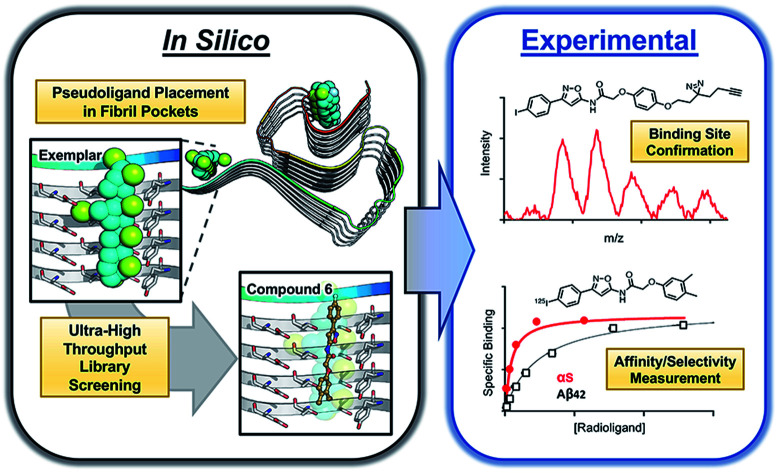
Ultra-high throughput *in silico* screening identified molecules that bind to α-synuclein fibrils, which were analyzed by photo-crosslinking, structure-activity studies, and radioligand binding to validate this approach for finding imaging probes.

## Introduction

α-Synuclein (αS) is a 140 amino acid, intrinsically disordered protein which is abundantly expressed at the presynaptic termini of central nervous system neurons.^1^,^2^ When bound to membranes, αS takes on a partially helical structure.^3^,^4^ Although the function of αS is not fully understood, its localization, along with knockout and overexpression studies, suggest that alongside synapsin, VAMP2 and others, αS plays a role in maintaining synaptic vesicle reserve pools, neurotransmitter release and synapse function and plasticity.^1^,^5^,^6^ Conversely, the pathological role of αS is well documented, where neuronal inclusions, comprised principally of β-sheet rich fibrillar αS termed Lewy bodies (LBs) and Lewy neurites (LNs), have long served as a post-mortem hallmark of Parkinson's disease (PD).^7^,^8^ Furthermore, similar aggregates can be observed in dementia with Lewy bodies (DLB), while a second form of aggregate has been identified in multiple system atrophy (MSA).^9^ Compared to LBs and LNs which occur in neurons of the substantia nigra, αS aggregates associated with MSA are found in oligodendrocytes of white matter tracts and are referred to as glia cell inclusions (GCIs).^9^ However, the inability to track fibril formation and localization in living patients has hindered the development of robust correlations between fibril distribution and PD, DLB, or MSA progression and prevents αS fibrils from serving as useful clinical markers.

To date, diagnosis of PD has relied heavily on the presentation of clinical symptoms, primarily motor deficits. Although these symptoms are effective in tracking progression at later stages of these diseases, they are not observed until a substantial degree of neuronal loss has occurred.^10^ Moreover, these symptoms are not exclusive to PD, but are observed for other Parkinsonian syndromes.^11^ Therefore, since differences in the presence and localization of fibrillar αS have already been established, methods for tracking deposits in patients could clarify diagnosis. Over the past decade, a breakthrough in the clinical evaluation of Alzheimer's disease (AD) was enabled by *in vivo* imaging of protein deposits with positron emission tomography (PET).^12^–^18^ The development of specific amyloid β (Aβ) and tau PET probes has allowed researchers to determine that the formation of Aβ aggregates precedes disease onset while tau-based neurofibrillary tangles (NFTs) occur later in disease progression.^19^ These promising results from imaging studies in AD patients have generated interest in the development of PET tracers to image αS fibrils and improve the diagnosis of PD.

Despite these advancements, the development of imaging probes with the requisite specificity for use in PD research and diagnosis has been challenging. In addition to LBs and LNs, PD patients also frequently present neuronal aggregates comprised of Aβ and tau.^20^ Although our laboratories and others have successfully identified compounds with moderate binding affinities for employment as PET imaging agents, no compound to date has displayed sufficiently high affinity and specificity for use as a radioligand.^21^,^22^ Following publication of the first solid-state NMR (ssNMR) structure of αS fibrils by Rienstra and colleagues, we demonstrated that through the combined use of computational docking, competition radioligand binding assays, and photo-crosslinking mass spectrometry we were able to describe distinct binding sites for several previously developed compounds.^23^,^27^

Here, we explore the utility of another computational approach, exemplar-based *in silico* screening, in an effort to develop a molecule that potently and specifically binds to αS fibrils.^28^ Through the application of this method we have identified a molecular scaffold, confirmed its binding site through photo-crosslinking, and used structure–activity relationship (SAR) studies to identify members of this compound series that have nanomolar affinity for αS fibrils with moderate specificity for αS over Aβ fibrils and other neuronal proteins. We go on to demonstrate the potential for development of this molecule as a PET probe through binding αS deposits in mouse brain tissue using a radiolabeled analog of the identified molecule. Finally, we provide SAR analysis based on the ssNMR structure used in our original *in silico* screening as well as several cryo-electron microscopy (cryo-EM) structures that have become available since we initiated these studies.^29^–^33^

## Results

### Exemplar-based *in silico* screen

**Fig. 1:**
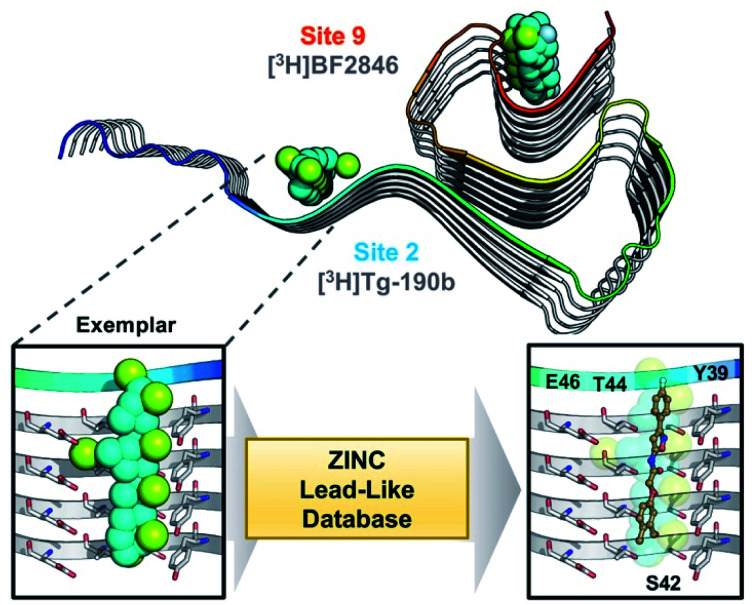
Exemplar-based *in silico* screening. (Top) Structure of αS fibril (PDB ID ; 2N0A) with exemplars for previously described Sites 2 (Y39-S42-T44) and 9 (G86-F94-K96) shown as spheres representing hydrophobic (cyan), hydrogen bond donating (yellow) and accepting (pale blue) pharmacophores. Compounds used in site-specific competition binding experiments are listed for each site in gray. (Bottom) Workflow for identifying small molecule binders.^23^ A zoom in on the Site 2 exemplar is shown on the left with compound **6** docked in the conformation identified from the ZINC 15 database using Align-It.^24^–^26^

Through methods developed by Karanicolas and colleagues, using the Rosetta Modeling Suite, exemplars can be facilely generated for any protein of interest given an input structure.^34^ An exemplar is a pseudoligand designed to be an ideal molecular complement to a surface exposed pocket on a protein of interest (Fig. 1).^28^,^34^ Following selection of an anchor residue, the protein is cast onto a three-dimensional grid, and grid points that correspond to the protein pocket are “chemotyped” by the adjacent functional features on the protein. That is, exemplar grid points are assigned characteristics that are complementary to the protein surface based on whether the protein surface presents hydrogen bond donating or accepting moieties or a hydrophobic patch. The exemplar therefore represents perfect compatibility with the protein surface, but contains no chemical bonds. Subsequently, scored molecular alignment of compound structures from a database allows for ultra-high throughput *in silico* screening to identify molecular architectures that satisfy the chemical features (*i.e.* hydrogen bonding/accepting positions) captured by the exemplar.^28^

In order to assess the potential of using an exemplar-based approach for αS PET probe development, we targeted sites that had been identified through our prior efforts as the binding sites for existing αS fibril radioligands (Fig. 1).^23^ In particular, two compounds, [^3^H]**Tg-190b** and [^3^H]**BF2846** (ESI, Fig. S8†), selectively bind to Site 2 (residues Y39-S42-T44, confirmed through photo-crosslinking in Hsieh *et al.*^23^) and Site 9 (residues G86-F94-K96, confirmed through **ClX1** photo-crosslinking in ESI, Fig. S6†) respectively, and can be utilized for *in vitro* competition binding assays to screen compounds identified *in silico*. Therefore, we chose these two sites as our initial targets. Using the ssNMR structure (PDB ID ; 2N0A) deposited by Rienstra and colleagues, pocket templates were generated using residues 44 and 86 of the central strand of the fibril as anchor points for Sites 2 and 9 respectively. The exemplar for each site was screened using Align-It against ∼10 million commercially available, lead-like molecules from the ZINC15 database.^24^–^26^ For each site, the 50 molecules scored as having the best alignment to each exemplar pseudoligand were retained (ESI, Fig. S2 and S3†). From this set of *in silico* hits, **17** compounds were purchased based on considerations of scaffold diversity.

**Fig. 2:**
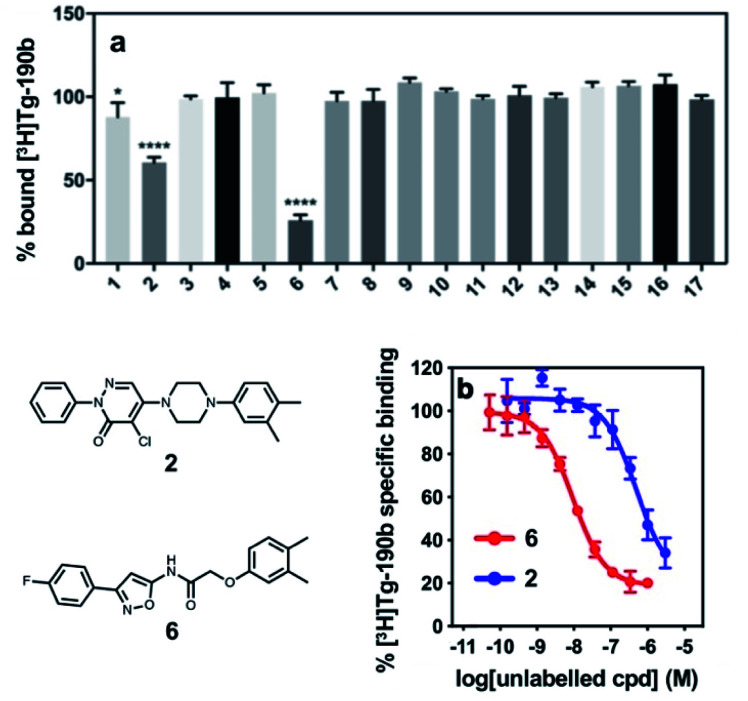
Competition binding assays of compounds from the exemplar-based screen. (a) Radioligand competition binding assay testing affinity of each compound (100 nM) against a Site 2 radioligand, [^3^H]**Tg-190b**, on αS fibrils. (b) Competition binding curves for αS fibrils (100 nM) with compounds **2** and **6**. αS fibrils were incubated with [^3^H]**Tg-190b** and increasing concentrations of competitors (**2** and **6**). Compounds **2** and **6** have IC_50_ values of 490 nM and 9.49 nM, respectively. Data points represent mean ± s.d. (*n* = 3). ANOVA was performed with Dunnett's multiple comparisons test, where *P < 0.0332, **P < 0.0021, ***P < 0.0002 and ****P < 0.0001.

These compounds were employed in a screening assay to determine their relative affinities for specific sites on αS fibrils. Of the set of molecules selected, two molecules were able to displace Site 2 radioligand [^3^H]**Tg-190b** (Fig. 2A). Competitive binding experiments revealed that compounds **2** and **6** displayed inhibitory concentrations (IC_50_s) of 490 nM and 9.49 nM, respectively, against the Site 2 radioligand (Fig. 2b).^35^

### Lead compound binds to site 2 and does not affect aggregation

Following the identification of two potential lead compounds, we decided to further investigate the more potent binder to confirm that the compound (1) binds to the target site on αS fibrils and that (2) the compound is non-perturbing and does not affect the aggregation of αS. We believe that a suitable imaging compound should not perturb the aggregation process, either by increasing or decreasing the amount of fibrils or altering their conformations. Therefore, a derivative of compound **6** was synthesized with a photo-crosslinking group (**ClX2**) to confirm the binding location (ESI, Scheme S2†). Following incubation of compound **ClX2** with αS fibrils and irradiation with 365 nm light, the sample was analyzed by whole protein and trypsin digest mass spectrometry (ESI, Fig. S7†). Analysis of the resulting data revealed a mass shift corresponding to crosslinking of one molecule of **ClX2**, suggesting that most of the compound binds at a single site. Furthermore, analysis of the digested sample confirmed that crosslinking could be observed on peptides corresponding to the target site, Site 2, initially used for the *in silico* screen (ESI, Fig. S7†).

To confirm that the probe does not affect the aggregation state or propensity of αS, we generated fluorescently labeled αS for use in fluorescence polarization (FP) assays. By attaching fluorescein-maleimide to a Y136C mutant αS construct, FP can be used to monitor fibril aggregation and gross conformational changes, as well as the compound's effect on these processes, as previously described.^36^,^37^ Compound **6** did not show any significant effect on the rate of aggregation or on the stability of αS fibrils (ESI, Fig. S4 and S5†).

### SAR screen to improve affinity/specificity

**Fig. 3:**
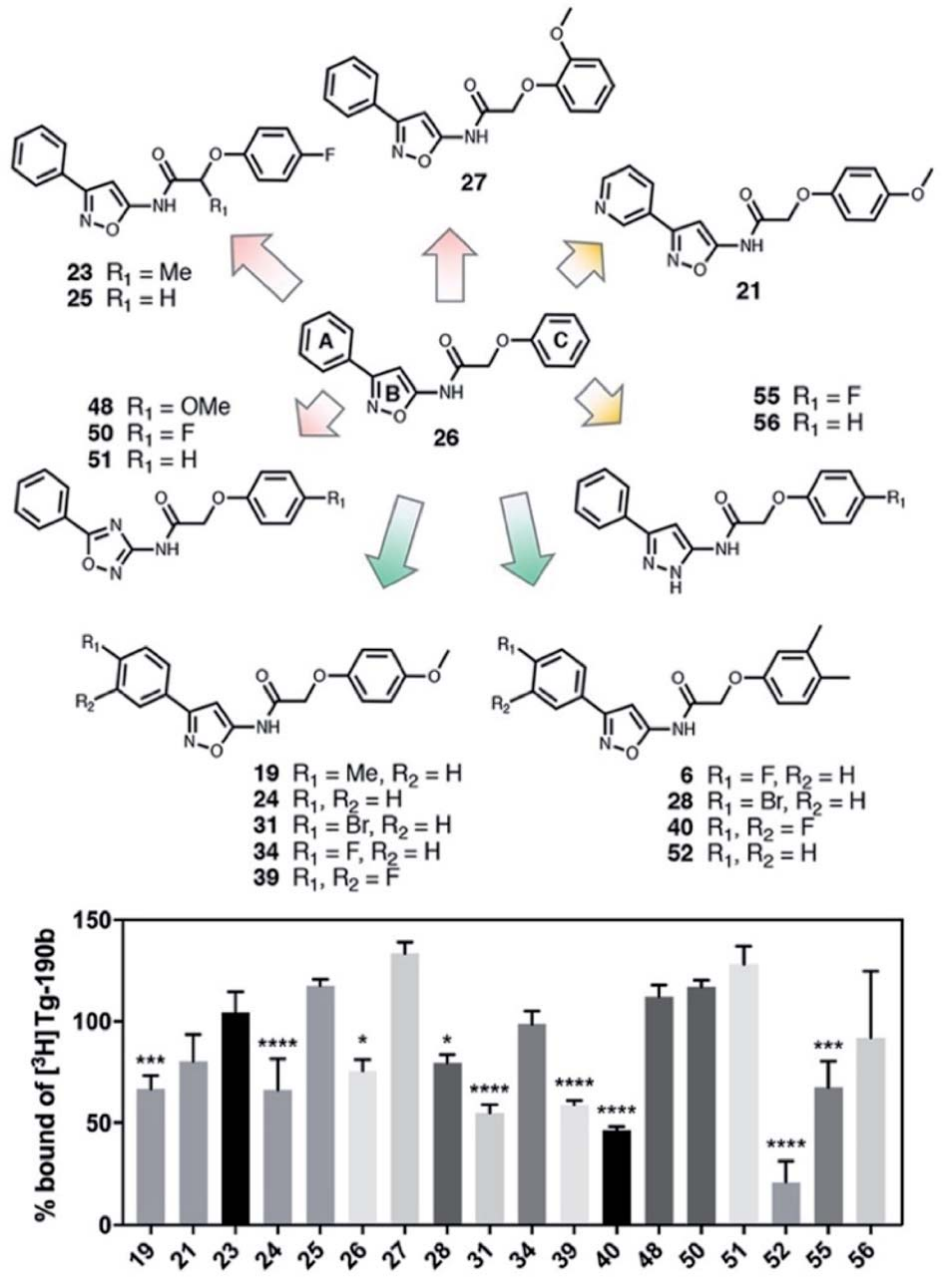
SAR screen based on exemplar lead compound **6**. (Top) Example set of compounds demonstrating SAR findings. Compound **26**, the base scaffold derived from compound **6**, is shown in the center with the A, B, and C ring systems highlighted. The compound set shown was selected to permit discreet analysis of substituent effects by direct comparison of two compounds (*e.g.*, **24***vs.***21** demonstrates the effect of heterocycle substitution of the A ring). Arrows indicate unfavorable (red), neutral (yellow), or favorable (green) modifications. (Bottom) Radioligand competition binding testing binding affinity of each compound shown against a Site 2 ([^3^H]**Tg-190b**) radioligand. Data points represent mean ± s.d. (*n* = 3). ANOVA was performed with Dunnett's multiple comparisons test, where *P < 0.0332, **P < 0.0021, ***P < 0.0002 and ****P < 0.0001. The full set of structures and binding data for all compounds tested in the SAR screen are given in ESI Fig. S9 and S10.†

In order to improve the affinity and selectivity of compound **6**, we conducted a SAR screen by culling structurally similar compounds from the Mcule library *via* a similarity search.^38^ In total, 39 molecules representing derivatives of the core scaffold **26** (Fig. 3, top) were used to determine the impact of various substituents on the A and C rings as well as replacement of the B ring with other 5-membered heterocycles. A subset of these compounds representing discreet changes to the core scaffold are shown in Fig. 3, along with their % bound of the Site 2 (Fig. 3, bottom) radioligand. The structures and competition binding data for the full set of compounds are shown in ESI Fig. S9 and S10.†

Several aspects of the SAR are clear. Changes to the A ring, particularly *para* substitution, are well-tolerated (**19**, **28**, **31**, **34**, **39**, **40**), and in many cases improve binding relative to molecules that are otherwise identical (**19** or **31***vs.***24**). Replacement of the A ring with a pyridine heterocycle (**21**) did not improve binding; nor did replacement with a furan or thiophene group (**20** and **22**, ESI, Fig. S9†), although these compounds lacked a direct comparator molecule. Compounds with oxadiazole (**48**, **50**, and **51**) replacements of the B ring all bound more poorly than the corresponding isoxazole compound (**48***vs.***24**), although pyrazole **55** had moderate affinity. Furthermore, we find that *ortho* substitutions of the C ring (**27** and ESI, Fig. S9,† **18**, **22**, **30**, **38**, **47**, **49** and **54**) hinder binding while *meta* and *para* substitutions (**19**, **24**, **28**, **31**, **34**, **39**, **40**, and **52**) can improve binding (**52***vs.***26**). However, the electronic nature of the C ring *para* substituent is important, as fluorinated compounds are weak binders (**25** and ESI, Fig. S9 and S10,† **32**, **35**, **37**, **44**, **50**), but moderate to strong binding is seen for compounds with electron donating methyl (**28**, **40**, and **52**) and methoxy (**19**, **24**, **31**, **34** and **39**) groups. Lastly, we find that compounds with substituents at the α-carbon of the central glycolic amide bind poorly (Fig. 3 **23** and ESI, Fig. S9† **35**), possibly due to a break in the planarity of the molecule.

Screening of the SAR library (Fig. S10†) was analyzed with one-way ANOVA and compounds showing the highest affinity for αS fibrils (*p* < 0.0001) were selected for in-depth characterization. For these compounds, we measured their affinity for both Site 2 and Site 9 in αS through multi-point competitive binding assays with [^3^H]**Tg-190b** and [^3^H]**BF2846** (Table 1). Compound **28** was found to have a higher affinity than compound **6**, with some apparent site-specificity.

**Table 1:** Comparison of IC_50_ values of select compounds for αS fibril sites^*a*^

Ligand	Site 2: [^3^H]**Tg-190b**	Site 9: [^3^H]**BF2846**
	IC_50_ (nM)	IC_50_ (nM)
**6**	9.49 (7.26–12.4)	109 (70.1–167)
**24**	95.1 (44.2–201)	557 (111–2279)
**28**	3.32 (2.06–53.1)	12.6 (7.00–22.9)
**31**	68.7 (27.1–169)	37.0 (25.6–53.5)
**39**	95.7 (50.4–182)	>1000
**40**	19.6 (7.13–53.9)	>1000
**52**	30.7 (17.0–56.8)	>1000

^*a*^Values were determined by competition binding assays with αS fibrils using radioligands [^3^H]**Tg-190b** or [^3^H]**BF2846**. 95% confidence intervals for IC_50_ values are shown in parentheses (*n* = 3).

### Synthesis and characterization of radioligand

**Scheme 1:**
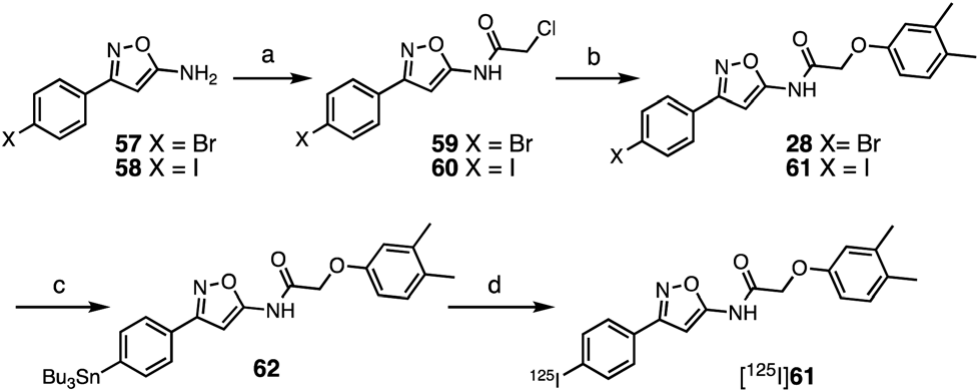
Synthesis of **61** and [^125^I]**61**. *Reagents and conditions*: (a) 2-chloroacetyl chloride, NET_3_, CH_2_Cl_2_, 0 °C–rt, 20 h (**59** 67%; **60** 65%); (b) 3,4-dimethylphenol, Cs_2_CO_3_, MeCN, 60 °C, 14–20 h, (**28** 36%; **61** 42%); (c) (SnBu_3_)_2_, Pd(PPh_3_)_4_, toluene, 110 °C, 3 h (55%); (d) [^125^I]NaI, H_2_O_2_, AcOH, MeOH, 60 min, 57% radiochemical yield, radiochemical purity >99%, molar activity of 81 GBq μmol^–1^.

The SAR studies and IC_50_ determinations identified compound **28**, a close analog of original hit **6**, as the most potent binder. Thus, we set out to synthesize and further characterize an iodinated derivative, compound **61**, as well as its radiolabeled isotopolog [^125^I]**61**. Chloroacetamide **60** was synthesized by reaction of iodo-isoxazolamine **58** and 2-chloroacetyl chloride, and subsequently coupled with 3,4-dimethylphenol to obtain iodo-derivative **61** with an overall yield of 27% (Scheme 1). In order to obtain the precursor for radioiodination, compound **28** was synthesized in a similar manner starting from bromo-isoxazolamine **57**, and subsequent stannylation of **28** yielded precursor **62**. Radioiodination was then successfully achieved at room temperature to obtain [^125^I]**61** for *in vitro* binding experiments.

**Fig. 4:**
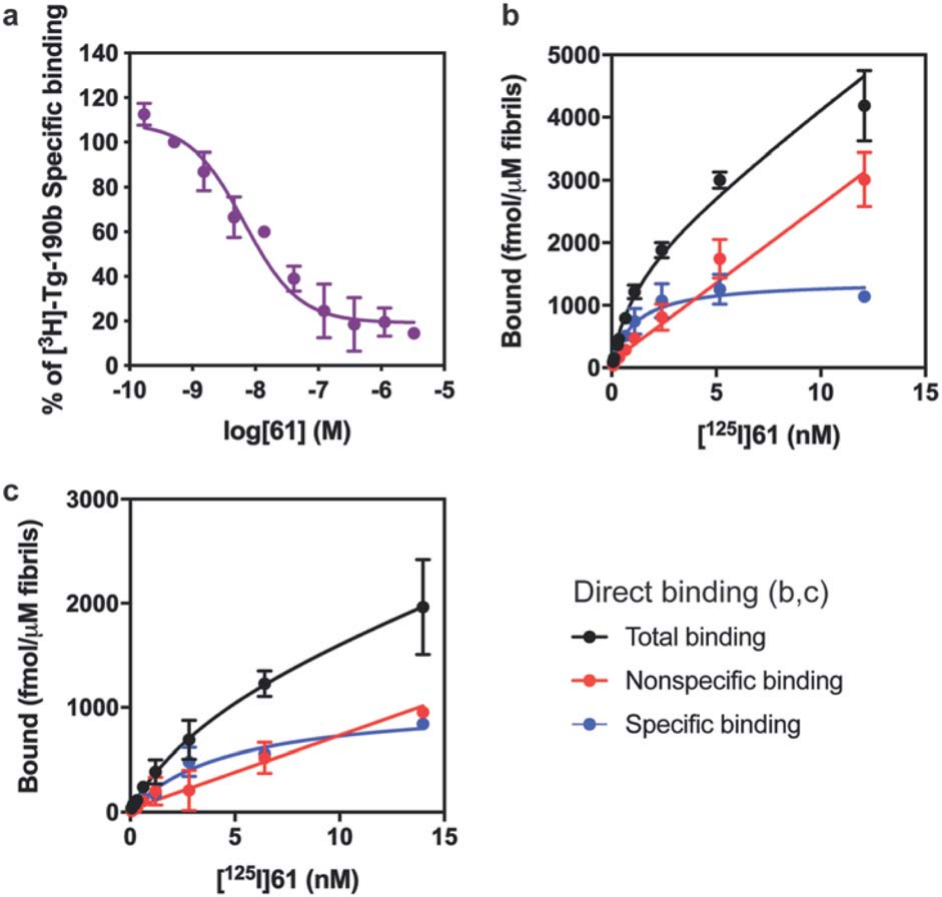
Compound **61** binds to αS fibrils with nM affinity. (a) Competition binding curves in αS fibrils with [^3^H]**Tg-190b**. (b) Compound **61** has an IC_50_ of 6.69 nM. (c) Saturation binding curves for [^125^I]**61**. *K*_d_ values were obtained for αS fibrils (b, *K*_d_ = 1.06 nM) and Aβ42 fibrils (c, *K*_d_ = 4.56 nM). Data points represent mean ± s.d. (*n* = 3).

First, we confirmed that replacement of the fluorine or bromine in compounds **6** and **28** with an iodine did not perturb the relative affinity for Site 2 (Fig. 4a) *via* competition binding experiments performed with [^3^H]**Tg-190b** and non-radioactive compound **61**. Saturation binding of [^125^I]**61** to αS fibrils confirmed the compound's high affinity, displaying a dissociation constant of 1.06 nM (Fig. 4b). We also demonstrated that **61** has a ∼5-fold selectivity (Fig. 4c) for αS fibrils over amyloid β (using fibrils of the 42 amino acid Aβ42 peptide). While these results are promising, it should be noted that the levels of non-specific binding were relatively high. Crosslinking with **CLX2** followed by labeling with a fluorescent azide demonstrated binding to αS fibrils, but also to many other proteins in mouse brain lysate (ESI, Fig. S13†).

**Fig. 5:**
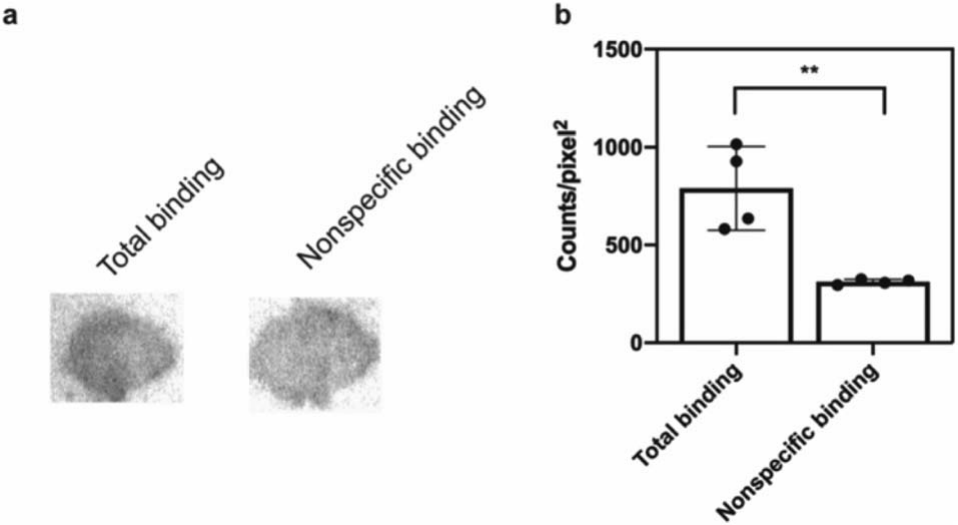
*In vitro* autoradiography on sarkosyl-insoluble fraction to assess [^125^I]**61** binding. (a) Autoradiograms showing the binding of [^125^I]**61** (1 nM) in sarkosyl-insoluble fraction from A53T mouse brain. Non-specific binding was defined using unlabeled **61** (100 nM). (b) Quantification of autoradiograms (*n* = 4); *t*-test was performed, where *P < 0.0332, **P < 0.0021, ***P < 0.0002 and ****P < 0.0001.

The potential efficacy of this radioligand as an imaging probe was then tested by *in vitro* autoradiography studies. Images were obtained by incubating 10 μm thick sections of sarkosyl-insoluble fraction from 15 month old PD mouse model (A53T) with [^125^I]**61** and subsequent exposure to storage phosphor screens. The autoradiograms and quantification thereof are shown in Fig. 5. The images clearly demonstrate binding of [^125^I]**61**, and this binding can be blocked by co-incubation with unlabeled **61**. Autoradiograms of the entire brain section from A53T and C6C3F1/J control mouse were also obtained (ESI Fig. S14†). While there is increased signal in A53T mouse brain in αS-rich regions compared with the control, the non-ideal physicochemical properties of this radioligand make it unsuitable to accurately assess binding on whole brain sections.

## Discussion

The development of a PET tracer to image PD is a high priority in the field of radiopharmaceutical research, since a radioligand that binds to αS could greatly improve the clinical diagnosis of PD. Despite significant academic and industrial effort in recent years, a PET tracer for αS with high affinity and selectivity has yet to be developed. In this work, we utilized a combination of exemplar-based *in silico* screening and radioligand binding studies to identify several compounds that bind to αS fibrils with nanomolar affinity and with moderate selectivity over Aβ fibrils. We are currently working to optimize **61** for *in vivo* PET studies. With this in mind, several aspects of our results are worth considering in more depth, including the implications of the SAR study for compound optimization, the importance of the ssNMR structure among various currently available αS fibril structures, and the relevance of models based on these structures to fibrils in tissue.

**Fig. 6:**
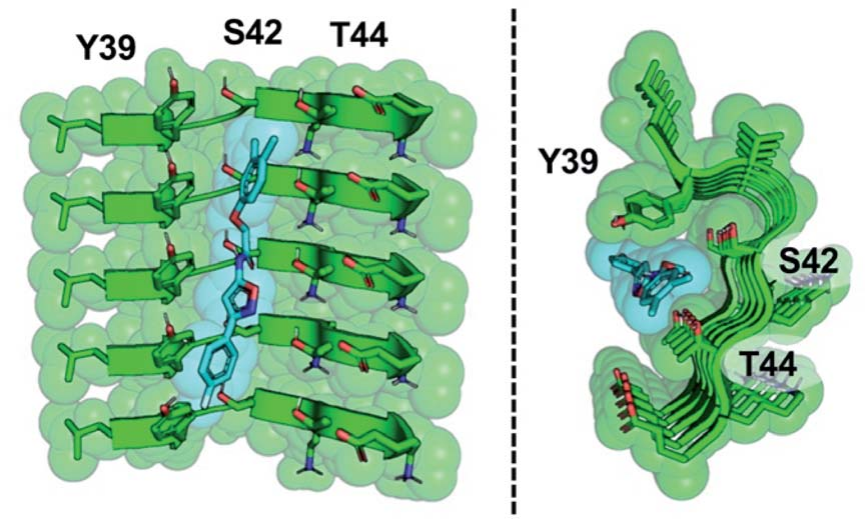
Model of compound **6** bound to Site 2. Two views of an equilibrated structure of compound **6** in the ; 2N0A Site 2 binding pocket, highlighting steric fit. Compound **6** was docked into the structure by alignment to the Site 2 exemplar and the structure minimized as described in ESI.†

The SAR study reveals that features of all three rings and the unsubstituted nature of the central amide unit are important for high affinity binding. An examination of docked compound **6** in the αS ; 2N0A structure (Fig. 6) reveals how several of these trends can be rationalized. The narrowness of the cavity is consistent with the fact that methyl substituents in compounds such as **23** generate non-planar structures which cause steric binding, *ortho* substituents weaken binding (as in **30**). These clash with the sidechains of Y39 and T44 (Fig. 6b). While *meta*, and especially *para*, substituents on the C ring improve binding, *ortho* substituents would clash with the protein backbone at K43/S42. On the A ring, *para* halogenation improves Site 2 affinity, but this seems to be a steric effect, rather than an electronic effect, since electron-donating methyl substituents (**19**) at this position are also tolerated and difluorination in **39** and **40** does not dramatically improve binding. However, it is possible that there are counterbalancing electronic effects on interactions of the A ring π system with the Y39 and T44 sidechains. Finally, the fact that the compounds with a 1,2,4-oxadiazole B ring do not bind well indicates that the SAR around this ring system is subtle and may depend on effects on the acidity of the heteroatoms, altering their hydrogen bonding ability with the T44 sidechain or even the backbone. Certainly, additional experiments with a tailored compound set will be necessary to fully understand the binding mode; we will also employ modeling that accounts for multiple rotamers and the various structures of αS fibrils that are now available.

**Fig. 7:**
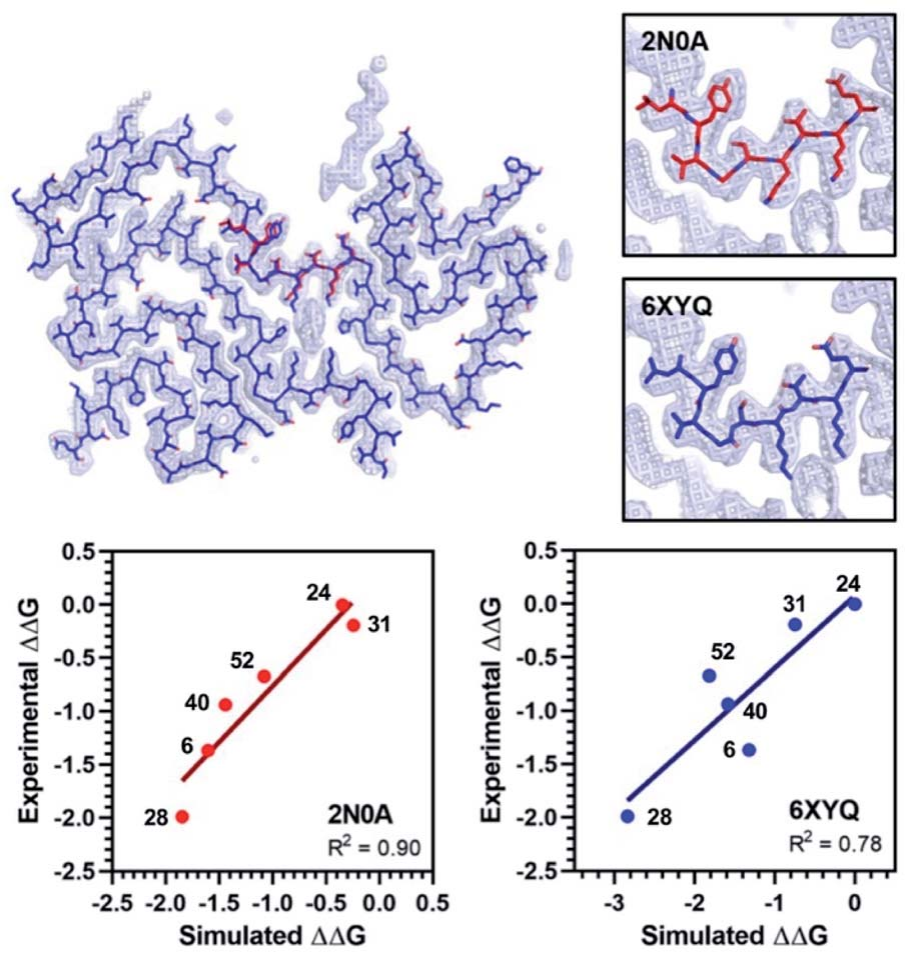
Comparison of 2N0A and ; 6XYQ Site 2 structures. (Top left) Overlay of aligned ; 2N0A and ; 6XYQ Site 2 coordinates with ; 6XYQ cryo-EM density map. (Top right) Overlay of ; 2N0A (upper) or ; 6XYQ (lower) Site 2 coordinates with ; 6XYQ cryo-EM density map. (Bottom) Correlation between experimental ΔΔ*G* (kcal mol^–1^) values and simulated ΔΔ*G* values (kcal mol^–1^) computed from PyRosetta TLS values as described in ESI† for simulation of the ; 2N0A (left) and ; 6XYQ (right) structures. Compound numbers are shown next to points.

The exemplar *in silico* screen was performed with the ; 2N0A ssNMR structure, which was the only αS fibril structure available at the time that this study was initiated. Since then, an additional ssNMR structure and several cryo-electron microscopy (cryo-EM) structures have become available, including structures of fibrils isolated from MSA patients.^29^–^33^ Furthermore, biochemical and biophysical techniques have also characterized molecular differences between *in vitro* fibrillar preparations and between patient samples from the major synucleinopathies.^39^,^40^ It is notable that the Site 2 fold is similar to ; 2N0A Site 2 in some of these structures, but different in others (Fig. 7 and ESI, Fig. S15/S16†). We modeled the binding of each Table 1 compound in ; 2N0A Site 2 using a standard Autodock 4.2 procedure or by alignment to the original exemplar using Shape-It followed by minimization in PyRosetta where the total ligand score (TLS) of each molecule was computed. We determined correlations between the simulated ΔΔ*G* values, computed from PyRosetta TLS values or Autodock binding energies (BEs), and ΔΔ*G* values derived from the experimental IC_50_ values (Fig. 7 and ESI Fig. S14†). We found that PyRosetta TLS correlated significantly better than Autodock BE (*R*^2^(TLS) = 0.90, *R*^2^(BE) = 0.55). In addition to superior correlation, PyRosetta provided an accurate ranking of relative affinities of the compound set, whereas Autodock only correctly predicted the highest and lowest affinity compounds (ESI, Table S1†). While the small size of the data set does not allow us to conclude that the TLS correlations can reliably predict the affinities of new compounds, we believe that they can provide evidence that a given binding site structure is a good model for interpreting SAR data.

We performed PyRosetta-based docking and TLS calculations for the compounds in Table 1 with the Site 2 pocket from six of the available αS fibril structures. For these alternative structures, one can see that some, but not all, provide a good correlation between ΔΔ*G* values derived from the IC_50_ values and their calculated Site 2 TLS (ESI, Fig. S16†). The variability of these correlations indicates that some of the structures are better models for interpreting SAR data than others. In particular, one of the MSA patient-derived structures (; 6XYQ) shows a correlation comparable to ; 2N0A with a similar rank ordering of ligands. In spite of a somewhat different overall fold, the alignment of the Site 2 region for the ; 6XYQ (MSA) and ; 2N0A (ssNMR) structures is quite good as one can see from an alignment of the two sets of PDB coordinates and an overlay of the coordinates with the cryo-EM density map (Fig. 7). This implies that the ; 2N0A structure in the Site 2 region may be representative of αS fibrils in MSA patients and raises the exciting possibility that our compounds will have similar affinities in MSA patient tissue.

We expect that multiple patient-derived cryo-EM αS fibril structures will be reported, as they have been for tau and Aβ, using material derived from human patients with Alzheimer's disease or related neuropathies.^41^–^43^ With such structures available, an essential question for drug design will be whether they accurately represent the conformation (or conformations assuming that varying polymorphs underlie differences in synucleinopathies, as seen for tau) adopted by fibrils *in vivo*. SAR studies performed with patient tissue using the compound classes identified by our approach can be used in conjunction with correlations like those reported here to determine which of the structures best represents the conformations of the fibrils *in vivo*. Subsequent optimization for *in vivo* PET imaging will certainly focus on improvement of solubility, removal of probable metabolic liabilities, blood–brain barrier penetration, and selectivity against Aβ and tau fibrils. While some of this optimization will rely on empirical knowledge of the pharmaco-kinetic properties of functional groups, it will also be guided by computational modeling of candidate compounds bound to the most relevant structures of αS fibrils as well as counter-selection using structures of Aβ and tau fibrils.

In summary, we have used an ultra-high throughput *in silico* screen to identify lead compounds for PET imaging of αS fibrils in PD and related synucleinopathies. Lead compound **61** has a 1 nM affinity for αS fibrils with demonstrated animal tissue binding, and the correlations shown in Fig. 7 imply that our compound class may be particularly suited for imaging in MSA patients. Moreover, our approach is unique and represents a new paradigm for radioligand development. The exemplar strategy is particularly well-suited to the problem of identifying ligands for shallow surfaces where no existing binding partner or enzyme substrate can provide a starting point for molecular design. Future implementations of these methods will also include pre-screening the compound library to remove compounds with unfavorable physico-chemical properties, which could help to avoid non-specific binding of the sort observed for compound **61**. This computational approach, coupled with screening of commercially available compounds in competition assays with radioligands, allows one to rapidly develop SAR for a given binding site. For a case such as the αS fibril, with multiple shallow binding sites, confirmation of site-specificity using photo-crosslinking also plays an important role. While we had previously established a set of site-specific ligands for competition assays, a *de novo* approach would also be possible in which *in silico* hits are converted to either radiolabeled or photo-crosslinkable compounds to establish binding sites and affinities. Indeed, the azidation reaction used to generate compound **ClX1** for confirming the Site 9 selectivity of **BF2846** (ESI, Fig. S6†) is nicely suited to this purpose. These initial lead compounds could then be used in high throughput assays with other unlabeled compounds as demonstrated in our work. Thus, the paradigm for imaging probe development established here should be broadly applicable to other fibril forming proteins, which are increasingly recognized as targets not just in PD, but also in other therapeutic areas with unmet molecular imaging needs such as amyotrophic lateral sclerosis (ALS) or neuroinflammation.

## Experimental

### Exemplar generation and *in silico* compound screen

The ZINC15, lead-like, commercially available compound database, consisting of ∼10 million molecules was used for the initial screen against the three sites.^24^ Exemplars were extracted from all residues in the PDB ; 2N0A ssNMR structure using default parameters in Rosetta and residues 156, 163 and 198 provided the best representations of the previously describes Sites 2, 3/13 and 9, respectively.^23^ Molecular alignments of target molecules to each selected exemplar were performed using Align-It which reduces each molecule/exemplar to a set of pharmacophores and reports a Tanimoto coefficient for each alignment, capturing both agreements in molecular features and their alignment in three-dimensional space.^25^ The top 30 compounds as quantified by the Tanimoto coefficient from each search were retained and a subset of compounds from each search was selected by hand for experimentation. Since the compounds identified for Site 3/13 were either too small or too similar to compounds previously explored,^23^ select compounds from the Site 2 and Site 9 screens were used in subsequent experimental screens and the full set of compounds can be found in ESI Fig. S2 and S3.†

### Screening compound library

Compounds **1–17** were purchased from vendors that were listed on the ZINC15 compound library. In order to screen the purchased compounds for αS binding, 100 nM of each compound was incubated for 1 h at 37 °C with 100 nM αS fibrils and [^3^H]**Tg-190b** (6 nM) or [^3^H]**BF2846** (3 nM) in 50 mM Tris–HCl, pH 7.4. Total binding was measured in the absence of competitor and nonspecific binding was determined in reactions containing unlabeled **Tg-190b** (1 μM) or **BF2846** (0.5 μM). After incubation, bound and free radioligand were separated by vacuum filtration, followed by washing with buffer containing 10 mM Tris–HCl (pH 7.4) and 150 mM NaCl. Filters containing the bound ligand were mixed with 3 mL of scintillation cocktail (MicroScint-20, PerkinElmer, Inc.) and counted after 12 h of incubation on a MicroBeta System (PerkinElmer, Inc.). All data points were measured in triplicate. Screening of compounds **18–56** for the SAR study was performed in a similar fashion.

### Saturation binding assay

αS (50 nM) or Aβ42 (100 nM) fibrils were incubated for 1 h at 37 °C with increasing concentrations of [^125^I]**61** in 50 mM Tris–HCl, pH 7.4, in a total volume of 150 μL. Nonspecific binding was determined in a duplicate set of binding reactions containing 2 μM non-radioactive **61**. After incubation, bound and free radioligand were separated by vacuum filtration through Whatman GF/C filters in a 24-sample harvester system, followed by washing with buffer containing 10 mM Tris–HCl (pH 7.4) and 150 mM NaCl. Filters containing the bound ligand were counted immediately on 2470 WIZARD Automatic Gamma Counter. All data points were collected in triplicate. The equilibrium dissociation constant (*K*_d_) and the maximal number of binding sites (*B*_max_) were determined by fitting the data to the equation *Y* = *B*_max_ × *X*/(*K*_d_ + *X*), using GraphPad Prism software.

### *In vitro* autoradiography on sarkosyl-insoluble fraction

The sarkosyl-insoluble fraction was obtained from brain homogenates of A53T (B6C3-Tg(Prnp-SNCA*A53T)83Vle/J) mice from The Jackson Laboratory. Frozen sections were thawed and incubated (1 h at room temperature) with 40% ethanol in PBS containing either [^125^I]**61** (1 nM) alone or [^125^I]**61** (1 nM) with 100 nM unlabeled **61**. After incubation, sections were washed in ice-cold 40% ethanol in PBS (3 × 1 min), followed by a wash in ice-cold DI water (1 min). Sections were dried in a stream of air, exposed to a storage phosphor screen (GE Healthcare) and the screen was imaged on a Typhoon FLA 7000 phosphor imager (GE Healthcare). Autoradiography images were quantified using Fujifilm Multi Gauge software.

### SAR correlation calculations

Two dimensional compounds were converted to three dimensional structures using openbabel.^44^ Compound **6** was aligned to each of the fibril architectures *via* their respective exemplars using Shape-It from Silicos-It.^26^ Derivatives were then aligned to the parent compound using Align-It, also from Silicos-It. Complexes were minimized using MinMover in PyRosetta where sidechain optimization was performed on the residues comprising Site 2 (residues 38–46), whereas backbone optimization was globally disallowed. Minimized complexes were scored using the beta_nov16 score function and the total ligand scores (TLSs) for the ligands in their minimized orientations were computed.^45^ Rosetta TLS values in REU were converted to ΔΔ*G* values in kcal mol^–1^ using the previously reported scaling factor of 2.94.^46^ Corresponding Autodock binding energy (BE) calculations were performed using a previously published protocol.^23^ Experimental ΔΔ*G* values were determined by using compound **52** as a reference. Computed ΔΔ*G* values from Autodock for the ; 2N0A structure and from PyRosetta for ; 2N0A, ; 6XYQ, and several other αS fibril structures were linearly regressed with the experimental ΔΔ*G* values to determine correlations. Details of all of these calculations are found in ESI† with comparisons of the other fibrils shown in Fig. S15/S16.†

## Conflicts of interest

There are no conflicts to declare.

## Supplementary Material

Supplementary information
