# Supplementary material for: Identification of a nanomolar affinity α-synuclein fibril imaging probe by ultra-high throughput in silico screening
Source: Chem Sci. 2020 Sep 10;11(47):12746–54. doi: 10.1039/d0sc02159h (PMC8047729; doi:10.1039/d0sc02159h)
Supplement: Supplementary file 1 [file SC-011-D0SC02159H-s001.pdf]

Electronic Supplementary Material (ESI) for Chemical Science.
This journal is © The Royal Society of Chemistry 2020

ELECTRONIC SUPPORTING INFORMATION

Identification of a Nanomolar Affinity α-Synuclein Fibril Imaging Probe by Ultra-High Throughput *In Silico* Screening

John J. Ferrie,[a,‡] Zsofia Lengyel-Zhand,[b,‡] Bieneke Janssen,[b] Marshall G. Lougee,[a] Sam Giannakoulias,[a] Chia-Ju Hsieh,[b] Vinayak Vishnu Pagar,[a] Chi-Chang Weng,[b] Hong Xu,[c] Thomas J. A. Graham,[b] Virginia M.-Y. Lee,[c] Robert H. Mach,[b] and E. James Petersson[a,*]

[a]Department of Chemistry, University of Pennsylvania, Philadelphia, Pennsylvania 19104, USA

[b]Department of Radiology, Perelman School of Medicine, University of Pennsylvania, Philadelphia, Pennsylvania 19104, USA

[c]Center for Neurodegenerative Disease Research, University of Pennsylvania, 3600 Spruce Street, Philadelphia, PA 19104, USA

Table of Contents:

1. ***In Silico* Screening Methods …………………………………………………...… S2**
2. **Synthetic Chemistry Methods…………………………………………………… S6**
3. **Protein Expression and Fibril Preparation……………..……………….……… S19**
4. **Fluorescence Polarization and Photo-Crosslinking Assays…………………..… S20**
5. ***In Vitro* Radioligand Binding Assays ……………………………...………… S25**
6. **Photo-crosslinking in Mouse Brain Lysate ……………………………...…. S33**
7. **Mouse Brain Radioligand Binding Assays ………………………….…….. S36**
8. **Computational Docking and Comparison of Fibril Structures………………….. S39**
9. **References……………………………………………………………….…. S45**

In Silico Screening Methods

Exemplar-based screening in Align-It. The Rosetta Modeling Suite was used to map exemplars at each residue of the 2N0A PDB by running[1]:

```
ROSETTA/make_exemplar.linuxgccrelease -database ROSETTA/database -in:file:s 2N0A.pdb -pocket_grid_size 12 -pocket_static_grid -pocket_filter_by_exemplar -pocket_surface_dist 1 -central_relax_pdb_num XX
```

Where residues for the previously identified sites 2, 3/13 and 9 corresponded to residues 156, 163 and 198.[2] In order to allow Align-It to recognize the exemplar outputs from Rosetta, the hydrogen bond donating and accepting parameters in Align-It were altered. Prior to compiling Align-It, the following lines were added at line 42 to the file hDonFuncCalc.cpp in the src directory:

```
if (a->GetAtomicNum() == 4)
{
    PharmacophorePoint p;
    p.func = HDON;
    p.point.x = a->x();
    p.point.y = a->y();
    p.point.z = a->z();
    p.hasNormal = false;
    p.alpha = funcSigma[HDON];
    pharmacophore->push_back(p);
}
```

Additionally, the following lines were added to line 42 to the file hAccFuncCalc.app in src directory:

```
if (atom->GetAtomicNum() == 10)
{
  if(_hAccCalcAccSurf(atom) < 0.02)
  {
    continue;
  }
  PharmacophorePoint p;
  p.func = HACC;
  p.point.x = atom->x();
  p.point.y = atom->y();
```

```
        p.point.z = atom->z();
        p.hasNormal = false;
        p.alpha = funcSigma[HACC];
        pharmacophore->push_back(p);
    }
```

The ZINC15, lead-like, commercially available compound database, consisting of ~ 10 million molecules was used for the initial screen against the three sites.[3] Molecular alignments of target molecules to each selected exemplar were performed using Align-It which reduces each molecule/exemplar to a set of pharmacophores and reports a Tanimoto Coefficient for each alignment which captures both agreements in molecular features and their alignment in three-dimensional space.[4] The top ~50 compounds as quantified by the Tanimoto Coefficient from each search were retained and a subset of compounds from each search was selected by inspection for experimentation. Since the compounds identified for site 3/13 were either too small or too similar to compounds previously explored,[2] select compounds from the site 2 and site 9 screens were used in subsequent experimental screens. The full set of compounds can be found in Figs. S2 and S3.

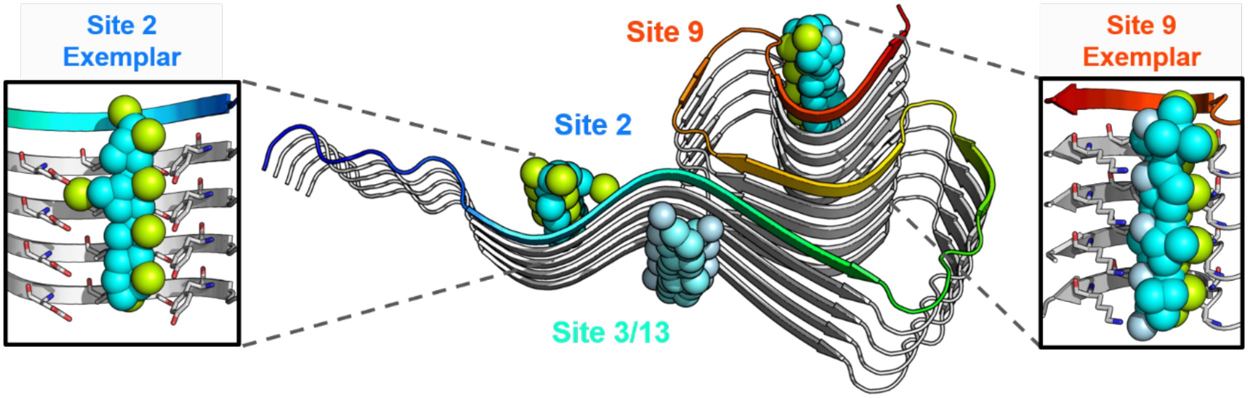

Fig. S1 Site 2 and Site 9 Exemplars Built from the PDB 2N0A Fibril Structure.[1] Exemplars for Sites 2 (Y39-S42-T44) and 9 (G86-F94-K96) shown as spheres representing hydrophobic (cyan), hydrogen bond donating (yellow) and accepting (pale blue) pharmacophores.

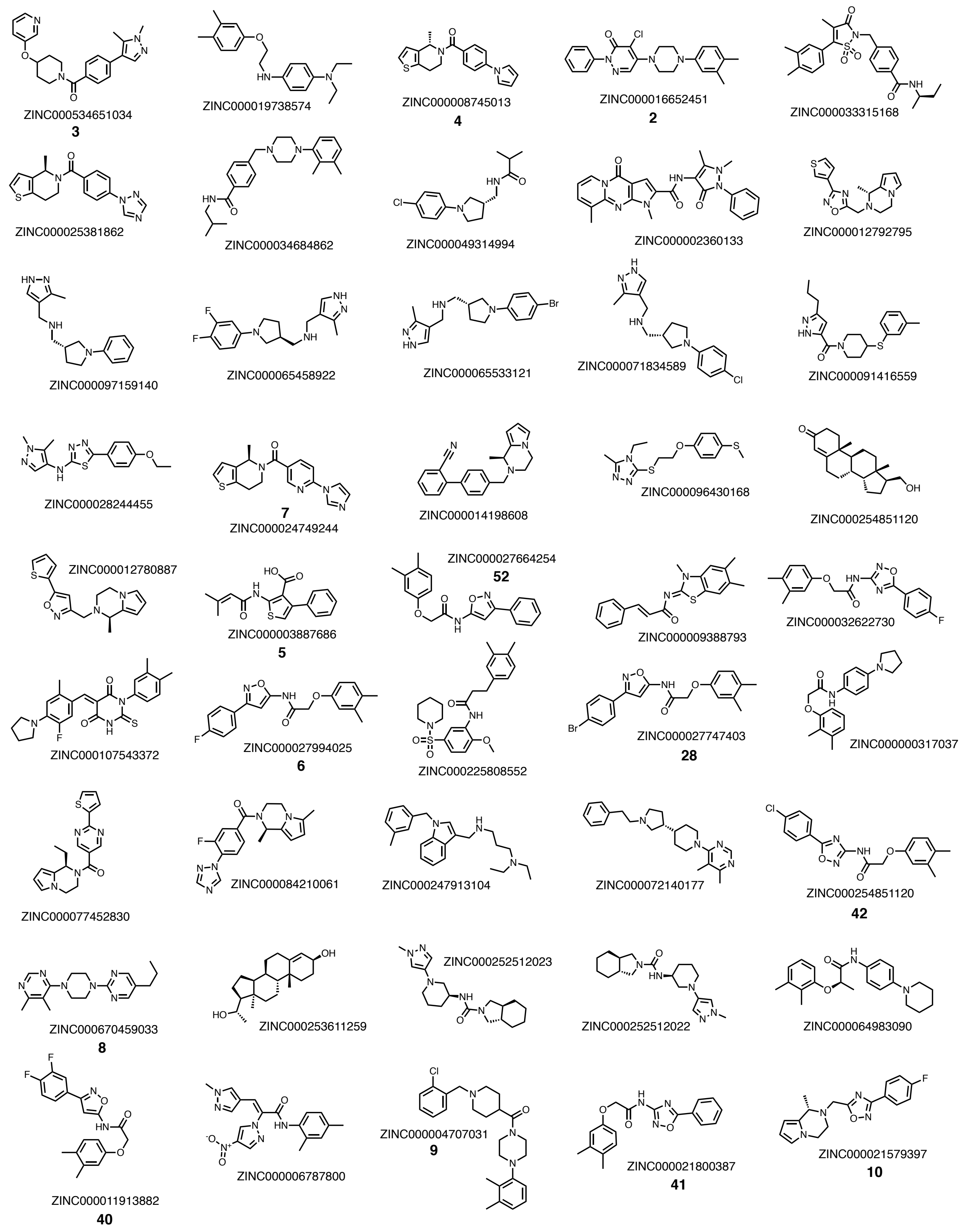

Fig. S2 Top Hits from ZINC15 Database for Site 2 Exemplar. Compounds are listed with their ZINC ID number. Compounds selected for screening are also listed with the number from the main text.

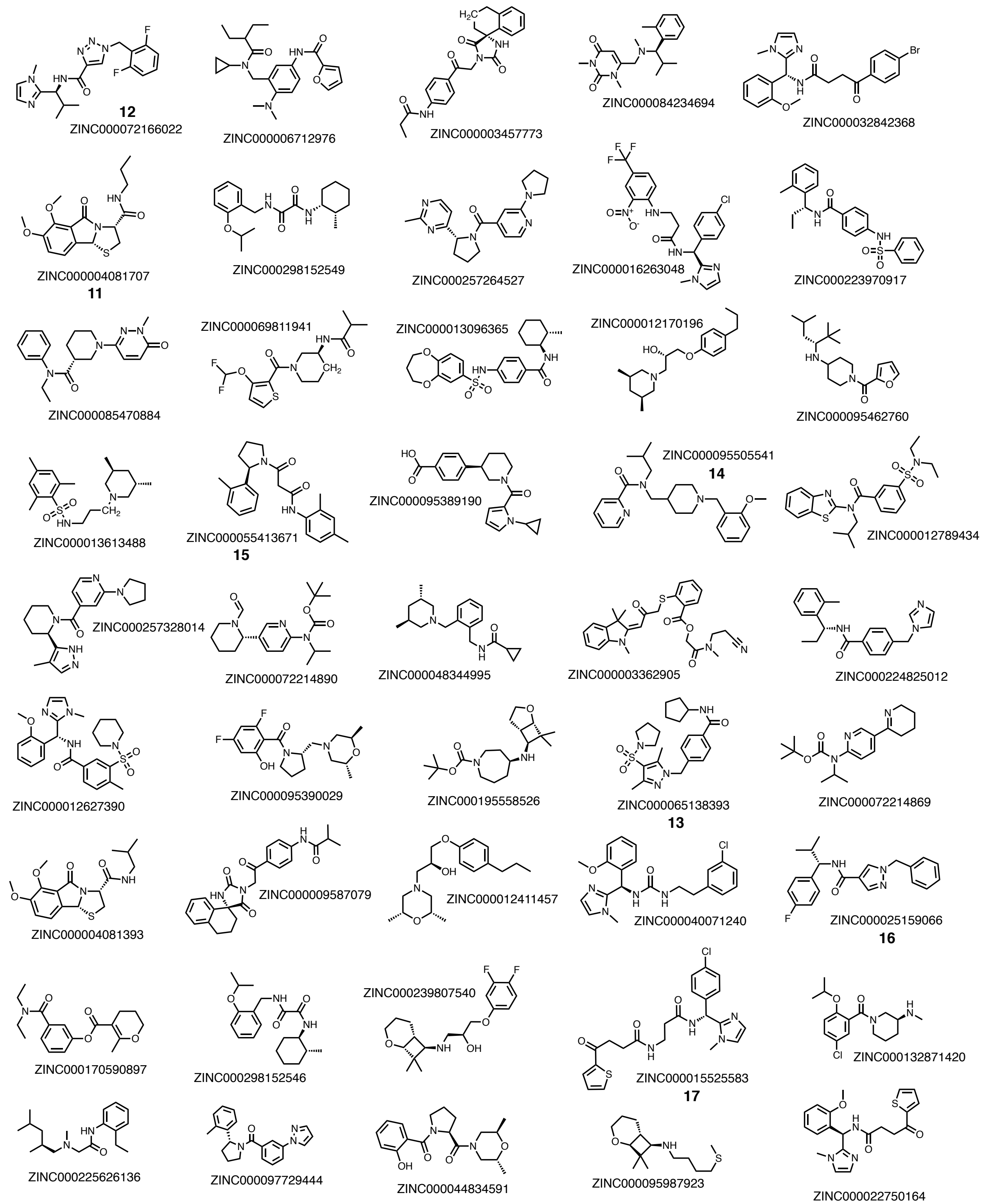

Fig. S3 Top Hits from ZINC15 Database for Site 9 Exemplar. Compounds are listed with their ZINC ID number. Compounds selected for screening are also listed with the number from the main text.

Synthetic Chemistry Methods

Chemical Reagents and Instruments. Chemicals were obtained from commercial sources and used without further purification. 3-(but-3-yn-1-yl)-3-(2-iodoethyl)-3*H*-diazirine was acquired from AstaTech Inc. Solvents were purchased from commercial sources and used as received unless stated otherwise. Reactions were performed at room temperature unless stated otherwise. Reactions were monitored by thin layer chromatography (TLC) on pre-coated silica 60 F254 aluminum plates (MilliporeSigma, Burlington, MA, USA), spots were visualized by UV light. Evaporation of solvents was performed under reduced pressure at 40 °C using a rotary evaporator. Flash column chromatography was performed on a Biotage® (Charlotte, NC, USA) Isolera One system equipped with Biotage® SNAP KP-Sil cartridges. Nuclear magnetic resonance (NMR) spectroscopy was performed on a Bruker (Billerica, MA, USA) Avance Neo 400 (400.17 MHz for ^{1}H and 100.63 MHz for ^{13}C) with chemical shifts (δ) reported in parts per million (ppm) relative to the solvent ($CDCl_3$, ^{1}H 7.26 ppm, ^{13}C 77.16 ppm; dimethyl sulfoxide (DMSO)-*d6*, ^{1}H 2.50 ppm, ^{13}C 39.52 ppm). Low resolution Liquid Chromatography Mass Spectrometry (LCMS) was carried out using a Waters (Milford, MA, USA) SQD equipped with an Acquity UPLC instrument in positive ion mode. High Resolution Mass Spectrometry (HRMS) for small molecules were obtained on a Waters LCT Premier XE LC/MS system. Matrix assisted laser desorption/ionization mass spectrometry (MALDI-MS) was performed on a Bruker Ultraflex III mass spectrometer.

Radioligand Synthesis

***N*-(3-(4-bromophenyl)isoxazol-5-yl)-2-chloroacetamide** (**59**). 2-chloroacetyl chloride (0.5 mL, 6.3 mmol) was added dropwise to a cooled (0 °C) solution of triethylamine (0.75 mL, 5.4 mmol) and 3-(4-bromophenyl)isoxazol-5-amine (500 mg, 2.1 mmol) in anhydrous CH_2Cl_2 (10 mL). The

mixture was stirred for 15 min at 0 °C, followed by 20 h of stirring at room temperature. The reaction was quenched with water (10 mL) while cooled on ice. The mixture was further diluted with water (20 mL) and CH_2Cl_2 (60 mL) and layers were separated. The organic layer was washed with saturated aqueous Na_2CO_3 (20 mL), dried over Na_2SO_4 and concentrated. The residue was subjected to flash column chromatography (gradient of 20-30% EtOAC/hexanes) to obtain *N*-(3-(4-bromophenyl)isoxazol-5-yl)-2-chloroacetamide **59** as a light tan solid (440 mg, 1.4 mmol, 67% yield).

TLC (hexanes:EtOAc, 70:30 *v/v*): R_f = 0.36; ^{1}H NMR (400 MHz, DMSO-d_6): δ 12.17 (s, 1H), 7.85-7.84 (m, 2H), 7.69-7.71 (m, 2H), 6.82 (s, 1H), 4.38 (s, 2H); ^{13}C NMR (100 MHz, DMSO-d_6): δ 163.61, 161.86, 161.75, 132.11, 128.60, 127.81, 123.77, 86.55, 42.78; LRMS (ESI positive ion mode, *m/z*): $[M+H]^+$ calcd. for $C_{11}H_9BrClN_2O_2^+$, 314.9530; found, 315.2; HRMS (*m/z*): $[M+H]^+$ calcd. for $C_{11}H_9BrClN_2O_2^+$, 314.9530; found, 314.9533.

***N*-(3-(4-iodophenyl)isoxazol-5-yl)-2-chloroacetamide** (**60**). 2-chloroacetyl chloride (0.25 mL, 3.0 mmol) was added dropwise to a cooled (0 °C) solution of triethylamine (0.35 mL, 2.5 mmol) and 3-(4-iodophenyl)isoxazol-5-amine (286 mg, 1.0 mmol) in anhydrous CH_2Cl_2 (7 mL). The mixture was stirred for 15 min at 0 °C, followed by 20 h of stirring at room temperature. The reaction was quenched with water (10 mL) while cooled on ice. The mixture was further diluted with water (20 mL) and CH_2Cl_2 (50 mL) and layers were separated. The organic layer was washed with saturated aqueous Na_2CO_3 (20 mL), dried over Na_2SO_4 and concentrated. The residue was subjected to flash column chromatography (gradient of 20-30% EtOAC/hexanes) to obtain *N*-(3-(4-iodoophenyl)isoxazol-5-yl)-2-chloroacetamide **60** as a light tan solid (235 mg, 0.65 mmol, 65% yield).

TLC (hexanes:EtOAc, 80:20 *v*/*v*): $R_f = 0.22$; ^{1}H NMR (400 MHz, DMSO-d_6): δ 12.16 (s, 1H), 7.87-7.89 (m, 2H), 7.66-7.68 (m, 2H), 6.80 (s, 1H), 4.38 (s, 2H); ^{13}C NMR (100 MHz, DMSO-d_6): δ 163.58, 162.02, 161.69, 137.91, 128.45, 128.05, 97.25, 86.46, 42.73; LRMS (ESI positive ion mode, *m/z*): $[M+H]^+$ calcd. for $C_{11}H_9ClIN_2O_2^+$, 362.9392; found, 363.3; HRMS (*m/z*): $[M+H]^+$ calcd. for $C_{11}H_9ClIN_2O_2^+$, 362.9392; found, 362.9391.

2-(3,4-dimethylphenoxy)-*N*-(3-(4-bromophenyl)isoxazol-5-yl)acetamide (**28**). A mixture of 3,4-dimethylphenol (142 mg, 1.2 mmol) and Cs_2CO_3 (357 mg, 1.1 mol) in anhydrous acetonitrile (MeCN; 2 mL) was stirred at room temperature for 1 h. A slurry of 2-chloro-*N*-(3-(4-bromophenyl)isoxazol-5-yl)acetamide **59** (190 mg, 0.6 mmol) in anhydrous MeCN (6 mL) was added and the resulting reaction mixture turned light brown. The reaction was heated to 60 °C and stirred for 20 h. The reaction mixture was diluted with CH_2Cl_2 (50 mL) and water (30 mL) and layers were separated. The water layer was extracted with CH_2Cl_2 (2x 25 mL) and combined organic layers were washed with saturated aqueous NH_4Cl (20 mL), dried over Na_2SO_4 and concentrated *in vacuo*. The crude compound was purified by flash column chromatography (gradient of 10-20% EtOAC/hexanes) to obtain 2-(3,4-dimethylphenoxy)-*N*-(3-(4-bromophenyl) isoxazol-5-yl)acetamide **28** as a light yellow solid (87 mg, 0.2 mmol, 36% yield).

TLC (hexanes:EtOAc, 80:20 *v*/*v*): $R_f = 0.33$; ^{1}H NMR (400 MHz, $CDCl_3$): δ 9.18 (s, 1H), 7.68-7.71 (m, 2H), 7.58-7.61 (m, 2H), 7.10 (d, $J = 8.3$ Hz, 1H), 6.79-6.80 (m, 2H), 6.72 (dd, $J = 2.8$ Hz, 8.3 Hz, 1H), 4.66 (s, 2H), 2.27 (s, 3H), 2.22 (s, 3H); ^{13}C NMR (100 MHz, $CDCl_3$): δ 165.11, 163.02, 159.92, 154.82, 138.68, 132.29, 131.21, 130.89, 128.42, 127.97, 124.73, 116.41, 111.74, 87.49, 67.36, 20.23, 19.03; LRMS (ESI positive ion mode, *m/z*): $[M+H]^+$ calcd. for

$C_{19}H_{18}BrN_2O_3^+$, 401.0495; found, 401.4 ;HRMS (*m/z*): $[M+H]^+$ calcd. for $C_{19}H_{18}BrN_2O_3^+$, 401.0495; found, 401.0509.

2-(3,4-dimethylphenoxy)-*N*-(3-(4-iodophenyl)isoxazol-5-yl)acetamide (**61**). A mixture of 3,4-dimethylphenol (134 mg, 1.1 mmol) and Cs_2CO_3 (361 mg, 1.1 mol) in anhydrous acetonitrile (MeCN; 2 mL) was stirred at room temperature for 1 h. A slurry of 2-chloro-*N*-(3-(4-iodophenyl)isoxazol-5-yl)acetamide **60** (198 mg, 0.5 mmol) in anhydrous MeCN (8 mL) was added and the resulting reaction mixture turned light brown. The reaction was heated to 60 °C and stirred for 14 h. The reaction mixture was diluted with CH_2Cl_2 (50 mL) and water (25 mL) and layers were separated. The water layer was extracted with CH_2Cl_2 (2x 25 mL) and combined organic layers were washed with saturated aqueous NH_4Cl (25 mL), dried over Na_2SO_4 and concentrated *in vacuo*. The crude compound was purified by flash column chromatography (gradient of 10-20% EtOAC/hexanes) to obtain 2-(3,4-dimethylphenoxy)-*N*-(3-(4-iodophenyl) isoxazol-5-yl)acetamide **61** as a light yellow solid (104 mg, 0.2 mmol, 42% yield).

TLC (hexanes:EtOAc, 80:20 *v*/*v*): R_f = 0.42; ^{1}H NMR (400 MHz, $CDCl_3$): δ 9.17 (s, 1H), 7.79-7.81 (m, 2H), 7.54-7.57 (m, 2H), 7.10 (d, J = 8.2 Hz, 1H), 6.79-6.80 (m, 2H), 6.72 (dd, J = 2.7 Hz, 8.2 Hz, 1H), 4.66 (s, 2H), 2.27 (s, 3H), 2.22 (s, 3H); ^{13}C NMR (100 MHz, $CDCl_3$): δ 165.11, 163.15, 159.93, 154.86, 138.68, 138.24, 131.22, 130.90, 128.56, 128.49, 116.44, 111.78, 96.64, 87.46, 67.41, 20.21, 19.02; LRMS (ESI positive ion mode, *m/z*): $[M+H]^+$ calcd. for $C_{19}H_{18}IN_2O_3^+$, 449.0357; found, 449.4; HRMS (*m/z*): $[M+H]^+$ calcd. for $C_{19}H_{18}IN_2O_3^+$, 449.0357; found, 449.0360.

2-(3,4-dimethylphenoxy)-*N*-(3-(4-(tributylstannyl)phenyl)isoxazol-5-yl)acetamide (**62**). A solution of 2-(3,4-dimethylphenoxy)-*N*-(3-(4-bromophenyl)isoxazol-5-yl)acetamide **28** (74 mg, 0.18 mmol), bis(tributyltin) (0.5 mL, 1.0 mmol) and $Pd(PPh_3)_4$ (42 mg, 0.04 mmol) in anhydrous toluene (6 mL) in a closed vial was purged with N_2 and heated to 100 °C for 3 h. The reaction mixture was concentrated *in vacuo* and the residue was subjected to flash column chromatography (10-15% EtOAc/hexanes) to obtain 2-(3,4-dimethylphenoxy)-*N*-(3-(4-(tributylstannyl)phenyl) isoxazol-5-yl)acetamide **62** as a light grey solid (62 mg, 0.10 mmol, 55% yield).

TLC (hexanes:EtOAc, 90:10 *v*/*v*): R_f = 0.23; ^{1}H NMR (400 MHz, $CDCl_3$): δ 9.14 (s, 1H), 7.74-7.76 (m, 2H), 7.54-7.56 (m, 2H), 7.10 (d, *J* = 8.3 Hz, 1H), 6.80 (m, 2H), 6.73 (dd, *J* = 2.7 Hz, 8.3 Hz, 1H) 4.66 (s, 2H), 2.27 (s, 3H), 2.22 (s, 3H), 1.51-1.58 (m, 12H), 1.27-1.38 (m, 6 H), 0.89 (t, *J* = 7.3 Hz, 9H); ^{13}C NMR (100 MHz, $CDCl_3$): δ 165.07, 159.52, 154.87, 145.66, 137.05, 130.87, 126.05, 116.44, 111.7875, 87.63, 67.43, 29.18, 27.46, 20.17, 18.98, 13.78, 9.76.

2-(3,4-dimethylphenoxy)-*N*-(3-(4-[^{125}I]iodophenyl)isoxazol-5-yl)acetamide ([^{125}I]**61**). 150 MBq (4.07 mCi) of [^{125}I]NaI solution in 0.1 M NaOH (PerkinElmer, Shelton, CT, USA), was added to 50 µL of a solution of stannyl precursor **62** (2.0 mg/mL solution in MeOH), directly followed by 50 µL of a pre-mixed solution of H_2O_2 (30%)/AcOH (1:3). The reaction mixture was vortexed and left to react at room temperature for 60 min, while shortly vortexed every 5-10 min. The reaction mixture was then diluted with HPLC mobile phase (1 mL, MeCN/50 mM NH_4CO_2 (pH 4.5) (80:20 *v*/*v*)) and subjected to preparative HPLC on an Agilent (Santa Clara, CA, USA) 1200 Series HPLC system equipped with a Luna C18 (5 µm, 100 Å, 250x10 mm) column (Phenomenex, Torrance, CA, USA) and Eckert & Ziegler (Hopkinton, MA, USA) FC-3300 radioactivity detector using the following gradient: 0-10 min 100% 0.1 M NH_4CO_2 (pH 4.5); 10-

30 min MeCN/0.1 M NH_4CO_2 (pH 4.5) (80:20, *v*/*v*); 30-65 min 100% MeCN (to elute unreacted precursor **62** at a flow rate of 3 mL·min^{-1}. The collected product fraction (t_R = 23 min, 10 mL collected) was diluted with H_2O (40 mL) and trapped on a solid phase extraction (SPE) cartridge (SepPak C18 Light, Waters, Milford, MA, USA) pre-conditioned with 1 mL of EtOH and 10 mL of H_2O. The SPE cartridge was washed with H_2O (10 mL) and [^{125}I]**61** (86 MBq, 2.33 mCi, 57% radiochemical yield) was eluted with ethanol (400 µL) for use in *in vitro* studies. Identity of the radiolabeled product was confirmed by co-injection of [^{125}I]**61** and unlabeled **61** on analytical HPLC (Agilent Eclipse XDB C18 5 µm, 4.6x150 mm column, MeCN/0.1 M NH_4CO_2 (pH 4.5) (70:30 *v*/*v*) at a flow rate of 1 mL·min^{-1}, t_R = 10 min). The radiochemical purity of [^{125}I]**61** was >99% with a molar activity of 81 GBq·µmol^{-1}.

BF2846 Photo-crosslinkable Analog (ClX1) Synthesis.

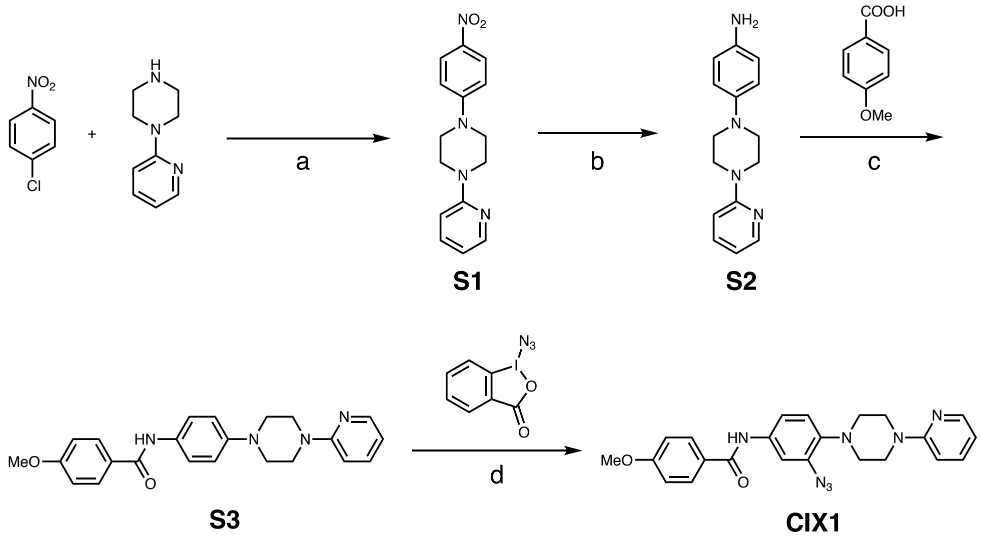

Scheme S1. Synthesis of **ClX1** Photo-crosslinker. a. $Pd_2(dba)_3$, RuPhos, NaO*t*-Bu, 100 °C, Dioxane, 78%; b. Pd/C, H_2, MeOH, room temperature, 15 h, 92%; c. HATU, DIPEA, DMF, room temperature, 6 h, 67%; d. $Rh_2(esp)_2$, MeOH, 50 °C, 7 h, 69%

Synthesis of 1-(4-nitrophenyl)-4-(pyridin-2-yl)piperazine (**S1**).[5] A sealed tube was charged with 1-chloro-4-nitrobenzene (500 mg, 3.17 mmol), 1-(pyridin-2-yl) piperazine (1.03 g, 6.3 mmol), Potassium tert-butoxide (609 mg, 6.3 mmol), Tris(dibenzylideneacetone) dipalladium(0) (73 mg, 0.079 mmol), and RuPhos (74 mg, 0.158 mmol). This mixture was subsequently treated with dioxane (10 mL). The sealed tube was closed and stirred vigorously at 100 °C for 20 min. The reaction progress was monitored by LCMS. The reaction mixture was cooled to room temperature and filtered through a Celite bed to remove any inorganic impurities. Solvent was removed under reduced pressure resulting in a crude residue, which was purified by flash chromatography on silica gel (eluting with 0-5% gradient of DCM / MeOH) to obtain 1-(4-nitrophenyl)-4-(pyridin-2-yl)piperazine (**S1**) (703 mg, 2.47 mmol, 78 %) as an orange solid.

TLC (DCM:MeOH, 95:5 v/v): R_f = 0.50; ^{1}H NMR (400 MHz, $CDCl_3$): δ 8.22-8.21 (m, 1H), 8.14 (d, J = 9.4 Hz, 2H), 7.52 (dd, J = 8.4, 5.4 Hz, 1H), 6.84 (d, J = 9.4 Hz, 2H), 6.68 (dd, J = 4.6, 1.4 Hz, 2H), 3.75 (t, J = 5.3 Hz, 4H), 3.58 (t, J = 5.3 Hz, 4H); ^{13}C NMR (100 MHz, $CDCl_3$): δ 158.9, 154.7, 148.2, 138.7, 137.8, 126.1, 114.0, 112.6, 107.5, 46.7, 44.6; HRMS-(ESI-TOF) (*m/z*): calcd, for $C_{15}H_{16}N_4O_2$ $[M+H]^+$ 285.1352; found 285.1376.

Synthesis of 4-(4-(pyridin-2-yl)piperazin-1-yl)aniline (**S2**). 1-(4-nitrophenyl)-4-(pyridin-2-yl)piperazine (690 mg, 2.42 mmol) was suspended in MeOH (10 mL) and 10% Pd-carbon (258 mg, 0.242 mmol) was added under an inert atmosphere at room temperature. The flask was carefully evacuated and a hydrogen balloon was inserted into the flask. The solution was then stirred for 15 h at room temperature. The reaction progress was monitored by LCMS. The reaction mixture was filtered through a Celite bed and washed with MeOH (2 x 15 mL). The filtrate was

then evaporated to obtain pure 4-(4-(pyridin-2-yl)piperazin-1-yl)aniline (**S2**) (567 mg, 2.22 mmol, 92%) as a light-yellow solid.

TLC (DCM:MeOH, 95:5 v/v): $R_f = 0.35$; ^{1}H NMR (400 MHz, $CDCl_3$): δ 8.21 (dd, J = 6.1, 1.3 Hz, 1H), 7.49 (td, J = 7.3, 1.9 Hz, 1H), 6.86 (d, J = 8.7 Hz, 2H), 6.69 (d, J = 8.8 Hz, 1H), 6.67 (d, J = 8.9 Hz, 2H), 6.63 (dd, J = 7.0, 3.5 Hz, 1H), 3.68 (t, J = 5.1 Hz, 4H), 3.47 (bs, 1.85H), 3.14 (t, J = 5.1 Hz, 4H); ^{13}C NMR (100 MHz, $CDCl_3$): δ 159.6, 148.1, 144.6, 140.6, 137.6, 119.1, 116.3, 113.6, 107.3, 51.2, 45.6; HRMS-(ESI-TOF) (*m/z*): calcd, for $C_{15}H_{18}N_4$ $[M+H]^+$ 255.1610; found 255.1606.

Synthesis of 4-methoxy -*N*-(4-(4-(pyridin-2-yl)piperazin-1-yl)phenyl)benzamide (**S3**). The 4-methoxybenzoic acid (97 mg, 0.639 mmol) and 1-[Bis(dimethylamino)methylene]-1H-1,2,3-triazolo[4,5-b]pyridinium3-oxide hexafluorophosphate (HATU, 243 mg, 0.639 mmol) were dissolved in dry dimethylformamide (DMF, 2 mL) at 0 °C. Diisopropylethylamine (DIPEA, 191 mg, 1.47 mmol) was added and the reaction mixture was stirred for approximately 5 min. The 4-(4-(pyridin-2-yl)piperazin-1-yl)aniline (125 mg, 0.491 mmol) was dissolved in DMF (1 mL) and added dropwise. The reaction was allowed to stir for 20 min before the ice was removed and the reaction was allowed to come to room temperature and then stirred for an additional 6 h. The reaction progress was monitored by LCMS. The reaction mixture was diluted with water (20 mL) and the aqueous phase was extracted with ethyl acetate (3 x 20 mL). The organic phase was washed with saturated NaCl solution (1 x 40 mL) and dried over Na_2SO_4. The solvent was removed under reduced pressure resulting in a crude residue, which was purified by flash column chromatography on silica gel (eluting with 0-5% gradient of DCM/ MeOH) to obtain 4-methoxy -*N*-(4-(4-(pyridin-2-yl)piperazin-1-yl)phenyl)benzamide (**S3**) (128 mg, 0.053 mmol, 67%) as an off white solid.

TLC (DCM:MeOH, 95:5 v/v): R_f = 0.15; ^{1}H NMR (400 MHz, DMSO-d_6): δ 9.91 (bs, 1H), 8.14 (dd, *J* = 4.8, 1.3 Hz, 1H), 7.95 (d, *J* = 8.8 Hz, 2H), 7.63 (d, *J* = 8.9 Hz, 2H), 7.56 (td, *J* = 8.7, 1.8 Hz, 1H), 7.05 (d, *J* = 8.8 Hz, 2H), 6.99 (d, *J* = 9.0 Hz, 2H), 6.90 (d, *J* = 8.6 Hz, 1H), 6.67 (dd, *J* = 6.8, 5.0 Hz, 1H), 3.84 (s, 3H), 3.64 (t, *J* = 5.0 Hz, 4H), 3.20 (t, *J* = 5.0 Hz, 4H); ^{13}C NMR (100 MHz, DMSO-d_6): δ 164.8, 162.2, 159.4, 148.1, 147.8, 138.0, 132.1, 129.9, 127.6, 121.9, 116.4, 114.0, 113.7, 107.8, 55.9, 49.2, 45.1; HRMS-(ESI-TOF) (*m/z*): calcd, for $C_{23}H_{24}N_4O_2$ $[M+H]^+$ 389.1978; found 389.1970.

Synthesis of *N*-(3-azido-4-(4-(pyridin-2-yl)piperazin-1-yl)phenyl)-4-methoxybenzamide (ClX1).[6] To an 8 ml vial, equipped with a septum screw cap, was charged with a magnetic stirrer bar, 4-methoxy -*N*-(4-(4-(pyridin-2-yl)piperazin-1-yl)phenyl)benzamide (30 mg,0.077 mmol), $Rh_2(esp)_2$ (2.3 mg, 0.0030 mmol), IBA-N_3 (45mg, 0.154 mmol), and MeOH (1.5 mL). The reaction was stirred at 55 ^{o}C for 5 h. The reaction progress was monitored by LCMS. The reaction mixture was concentrated and diluted with saturated bicarbonate solution (20 mL) and the aqueous phase was extracted with ethyl acetate (3 x 20 mL). The organic phase was washed with water (30 mL) and saturated NaCl solution (30 mL) and then dried over Na_2SO_4. Solvent was removed under reduced pressure resulting in a crude residue which was purified by flash column chromatography on silica gel (eluting with 0-5% gradient of DCM/ MeOH) to obtain *N*-(3-azido-4-(4-(pyridin-2-yl)piperazin-1-yl)phenyl)-4-methoxybenzamide (**ClX1**) (23 mg, 0.053 mmol, 69%) as a light-yellow solid.

TLC (DCM:MeOH, 95:5 v/v): R_f = 0.10; ^{1}H NMR (400 MHz, $CDCl_3$): δ 8.21 (dd, *J* = 4.9, 1.6 Hz, 1H), 7.83 (d, *J* = 8.8 Hz, 2H), 7.79 (bs, 1H), 7.57 (d, J = 2.3 Hz, 1H), 7.51 (td, *J* = 8.7, 1.8 Hz, 1H), 7.27 (dd, *J* = 8.5, 2.4 Hz, 1H), 7.01 (d, *J* = 8.6 Hz, 1H), 6.96 (d, *J* = 8.8 Hz, 2H), 6.70 (d, *J* =

8.6 Hz, 1H), 6.65 (dd, J = 6.9, 4.0 Hz, 1H), 3.87 (s, 3H), 3.73 (t, J = 4.9 Hz, 4H), 3.12 (t, J = 4.9 Hz, 4H); ^{13}C NMR (100 MHz, $CDCl_3$): δ 165.3, 162.7, 159.4, 147.8, 140.8, 137.9, 134.6, 133.9, 129.0, 126.9, 120.4, 117.4, 114.1, 113.6, 112.5, 107.4, 55.6, 51.6, 45.5; HRMS-(ESI-TOF) (*m/z*): calcd, for $C_{23}H_{23}N_7O_2$ $[M+H]^+$ 430.1991; found 430.1989.

Exemplar Lead Compound Photo-Crosslinkable Analog (ClX2) Synthesis

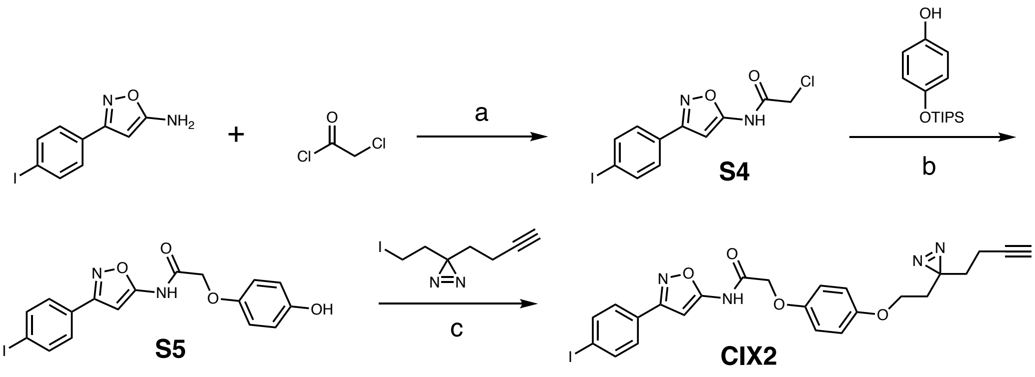

Scheme S2. Synthesis of ClX2 Photo-crosslinker. a. DMAP, NEt_3, DMF, 0 C, overnight, ~60%; b. Cs_2CO_3, DMF, 90 °C, overnight, 62%; b. Cs_2CO_3, DMF, 80 °C, overnight, 15%;

2-chloro-*N*-(3-(4-iodophenyl)isoxazol-5-yl)acetamide (**S4**). 3-(4-iodophenyl)isoxazol-5-amine (0.35 mmol, 100 mg, 1.0 equiv.) and 4-dimethylamino-pyridine (DMAP, 0.5 mol%) were charged to a flame-dried round bottom flask with a stir bar. The flask was sealed with a rubber septum and sparged with argon. Dimethylformamide (DMF, 3.0 mL) and trimethylamine (1.40 mmol, 111.5 μL, 4.0 equiv.) were then charged to flask, and the solution was cooled on ice for approximately 5 min. 2-chloroacetyl chloride (1.45 mmol, 202 μL, 4.1 equiv.) was then added dropwise. The reaction promptly turned dark brown/black, and was allowed to stir for 30 min before the ice was removed and the reaction was allowed to come to room temperature and stir overnight. The reaction was taken up in minimal ethyl acetate and transferred to a separatory funnel. The crude product was then washed with 1 M HCl, and the aqueous phase was extracted 3 times with ethyl acetate. The organic phase was then washed 2 times with 1 M HCl and 2 times with saturated

brine solution. The corresponding brown solid was adsorbed onto silica gel, loaded onto a 10 g normal phase silica hand-packed Biotage column, and eluted at a flow rate of 10 mL/min with EtOAc in hexanes (See gradient below). The product was obtained was an off-white powder in low to moderate yields of 56-63%.

Start (percent EtOAc)	End (percent EtOAc)	Column Volumes
5%	5%	10
5%	13%	15
13%	15%	15
15%	40%	10
40%	100%	5

TLC (hexanes:EtOAc, 50:50 *v*/*v*): R_f = 0.45 ^{1}H NMR (500 MHz, DMSO-d6) δ 12.14 (s, 1H), 7.88 (d, J = 8.5 Hz, 1H), 7.67 (d, J = 8.5 Hz, 2H), 6.80 (s, 1H), 4.38 (s, 2H). ^{13}C NMR (101 MHz, DMSO) δ 164.05, 162.49, 162.16, 138.38, 128.92, 128.53, 97.73, 86.93, 43.21. HRMS-(ESI-TOF) (*m/z*): calcd, for $C_{11}H_8ClIN_2O_2$ $[M+Na]^+$ 362.9392; found 362.9377.

***N*-(3-(4-iodophenyl)isoxazol-5-yl)-2-(4-((triisopropylsilyl)oxy)phenoxy)acetamide (S5)**

4-((Triisopropylsilyl)oxy)phenol (0.414 mmol, 120 mg, 3.0 equiv.) was solubilized in DMF (2 mL) and added to a dry round bottom flask followed by Cs_2CO_3 (0.552 mmol, 180 mg, 4.0 equiv.). The flask was then sealed with a rubber septum and sparged with argon. Compound **S4** (0.138 mmol, 50 mg, 1.0 equiv.) was then solubilized in DMF (2 mL) and added to the solution. The flask was then heated at 90°C overnight, resulting in conversion to product as well as deprotection of the triisopropyl silyl ether. The resulting product was taken up in 1 M HCl and extracted 3 times with EtOAc. The organic layer was washed 2 times with 1 M HCl, and 1 time with saturated

brine solution. The corresponding solid was then adsorbed onto silica gel, loaded onto a 10 g normal phase hand-packed Biotage column, and eluted at a flow rate of 10 mL/min with EtOAc in hexanes (See gradient below). The product (**S5**) was obtained as a fine reddish solid in 62% yield.

Start (percent EtOAc)	End (percent EtOAc)	Column Volumes
5%	5%	10
5%	23%	14
23%	26%	20
26%	100%	20

TLC (hexanes:EtOAc, 50:50 *v*/*v*): $R_f = 0.33$ ^{1}H NMR (500 MHz, DMF-d7) δ 12.17 (s, 1H), 9.38 (s, 1H), 8.13 (d, J = 8.4 Hz, 2H), 7.93 (d, J = 8.4 Hz, 2H), 7.11 (d, J = 8.9 Hz, 2H), 7.06 (s, 1H), 6.99 (d, J = 8.9 Hz, 2H), 5.00 (s, 2H). ^{13}C NMR (101 MHz, DMF) δ 166.44, 162.32, 152.63, 151.06, 138.28, 128.93, 128.57, 115.92, 115.83, 96.41, 86.54, 67.89.

2-(4-(2-(3-(but-3-yn-1-yl)-3*H*-diazirin-3-yl)ethoxy)phenoxy)-*N*-(3-(4-iodophenyl)isoxazol-5-yl)acetamide (ClX2)

Compound **S5** (0.060 mmol, 25 mg, 1.0 equiv.) was solubilized in 2 mL DMF, and was transferred to a dry round bottom flask. Cs_2CO_3 (0.190 mmoles, 62 mg, 3.1 equiv.) was then added to this solution, resulting in a dark blue solution. The solution was then sparged with argon, and 3-(but-3-yn-1-yl)-3-(2-iodoethyl)-3*H*-diazirine (0.180 mmol, 45 mg, 3.0 equiv.) was then transferred to vessel in 1 mL DMF. The reaction was heated at 80°C overnight. After cooling, the reaction mixture was taken up in EtOAc and transferred to a separatory funnel. The organic layer was then washed with 1 M HCl, and the aqueous phase was extracted 3 times with EtOAc. The resulting

organic phase was then washed 1 time with 1 M HCl and 1 time with saturated brine solution. The EtOAc layer was then concentrated under reduced pressure, and purified using silica column chromatography. (15% EtOAc in hexanes) A light yellow powder was obtained in low yield of 11%.

TLC (hexanes:EtOAc, 80:20 *v/v*): $R_f = 0.38$ ^{1}H NMR (500 MHz, DMSO-d6) δ 11.95 (s, 1H), 7.88 (d, J = 8.4 Hz, 2H), 7.67 (d, J = 8.4 Hz, 2H), 6.93 (d, J = 9.2 Hz, 1H), 6.88 (d, J = 9.2 Hz, 2H), 6.80 (s, 1H), 4.76 (s, 2H), 3.76 (t, J = 6.1 Hz, 2H), 2.83 (t, J = 2.7 Hz, 1H), 2.04 (td, J = 7.4, 2.7 Hz, 2H), 1.85 (t, J = 6.1 Hz, 2H), 1.65 (t, J = 7.4 Hz, 2H). ^{13}C NMR (126 MHz, DMSO) δ 166.39, 162.42, 162.27, 153.22, 152.30, 138.37, 128.90, 128.60, 116.09, 115.84, 97.69, 86.96, 83.68, 72.27, 67.56, 63.31, 32.31, 32.25, 27.53, 13.12.

HRMS (m/z): $[M+H]^+$ calcd. for $C_{24}H_{21}IN_4O_4$, 557.0686; found, 557.0676.

Protein Expression and Fibril Preparation

Preparation of wild-type α-synuclein (αS) monomer and αS fibrils. Recombinant expression and purification of wild-type α-synuclein (αS) protein was performed as previously described.[7] Fibrils were prepared in a manner similar to that previously described, where 100 μM αS monomer was incubated in 50 mM tris 150 mM NaCl, 0.05% NaN_3, pH 7.4 at 37 °C and shaken at 1300 rpm for 3 days using a Fisher Scientific Mixer.[7]

Preparation of Aβ42 fibrils. Aβ42 fibrils were prepared as described previously.[8] Briefly, monomer Aβ42 (1 mg, Bachem; Torrance, CA, USA) was dissolved in hexafluoroisopropanol (HFIP) at a concentration of 2 mg/mL and incubated for 1 h at 37 °C until the peptide completely dissolved. Then HFIP was evaporated under air. The peptide powder was dissolved again in HFIP (2 mg/mL), aliquoted and left to dry overnight under vacuum. Aliquots were stored in a freezer at -20 °C.

To prepare fibrils, the HFIP-treated peptide aliquot was dissolved in 10 mM NaOH (10 μL) solution, then the sample was diluted with 90 μL 10 mM phosphate buffer, pH 7.4. The concentration of the peptide solution was confirmed by measuring the absorbance at 214 nm with a NanoDrop 2000c spectrophotometer (ThermoFisher). The extinction coefficient (76848 $M^{-1}cm^{-1}$) was calculated using literature values.[9] Next, the solution was agitated by a continuous slow rotation at room temperature for 3 days and fibril formation was confirmed by TEM. Briefly, samples (5 μL) were spotted onto glow-discharged formvar/carbon-coated, 200-mesh copper grids (Ted Pella). After 1 min, grids were washed briefly with water and stained with one 10 μL drop of 2% w/v uranyl acetate for 1 min. The excess stain was removed by filter paper and the grids were dried under air. Samples were imaged with a Tecnai FEI T12 electron microscope at an acceleration voltage of 120 kV. Images were recorded on a Gatan OneView 4K Cmos camera.

Fluorescence Polarization and Photo-Crosslinking Assays

Aggregation and disaggregation fluorescence polarization experiments. Wild-type and αS with a fluorescein maleimide attached via Cys114 mutation were produced as previously described.[10] Aggregations were performed at a total monomer concentration of 100 μM with 1% labeled αS in 20 mM Tris, 100 NaCl pH 7.4 in the presence of 10 and 100 μM compound **6** and with equivalent volume of DMSO as a control. At each timepoint, an aliquot of the fibrilization reaction was removed and diluted 10-fold in buffer. Fluorescence polarization (FP) was measured in a Greiner black 96 half-area well microplate (Greiner Bio-One North America Inc.; Monroe, NC, USA) at a total volume of 50 μL on a Tecan F200 plate reader (San Jose, CA, USA). Aggregations were performed in triplicate. No significant effect on aggregation rates was observed. These data are shown in Fig. S4.

Disaggregation experiments were performed on labeled fibril samples prepared as described above in the absence of compound. A final concentration of 10 μM fibrils was added to each well for each disaggregation experiment and compound **6**, nordihydroguaiaretic acid (NDGA), epigallocatechin gallate (EGCG) or DMSO (1%) was added just prior to measurement to the final concentrations detailed in Fig. S3 and total volumes of 50 μL. Samples were shaken at 149 rpm at room temperature with FP measurements taken every 150 s. EGCG and NDGA disrupted fibril stability as previously reported,[11] but compound **6** had no apparent effect (Fig. S5).

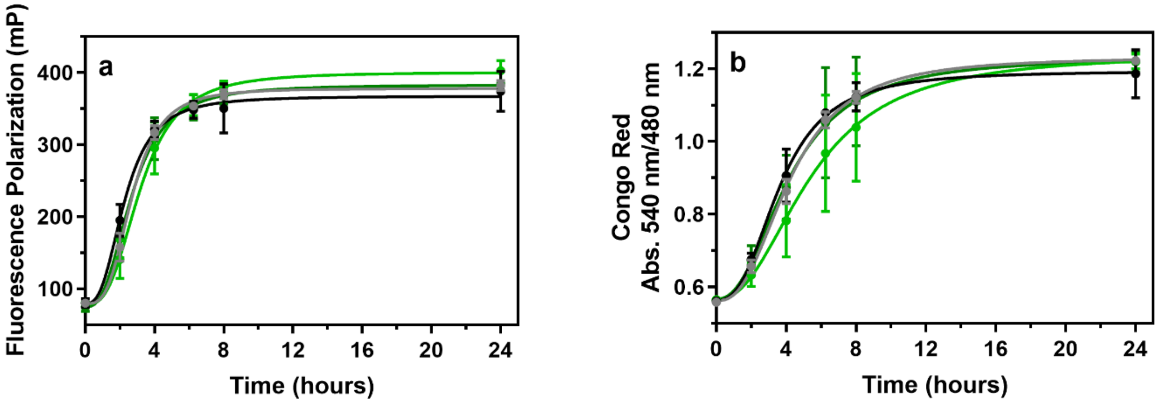

Fig. S4 Impact of Compound **6** on αS Aggregation. Aggregation of αS in the presence of 1% labeled monomer along with (light-green) 10 and (dark-green) 100 μM compound **6** monitored by (a) fluorescence polarization (FP) and (b) Congo Red absorbance. Aggregation in the presence of compound was compared to aggregation in the presence of volume matched addition of DMSO for (grey) 10 and (black) 100 μM compound administration.

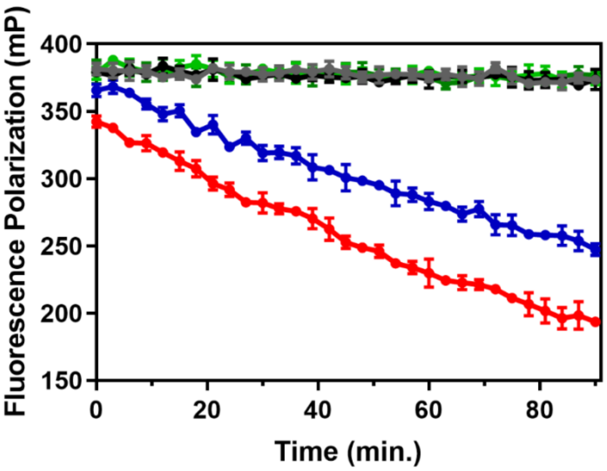

Fig. S5 Impact of Compound **6** on αS Fibril Stability. Assessment of potential remodeling of 1% labeled αS fibrils by (light-green) 10 and (dark-green) 100 μM compound **6** compared to (red) 100 μM EGCG and (blue) 100 μM NDGA as a positive control. DMSO administration volume-matched to that used for (grey) 10 and (black) 100 μM compound administration was used as a negative control.

Photo-crosslinking assay. Aggregations of αS prepared as described above were transferred to a clear glass vial to assist in photo-crosslinking, and compounds **ClX1**, **ClX2**, or DMSO (as a vehicle control) were delivered to their respective vials (100 μM final concentrations). The resulting fibril-crosslinker or fibril-DMSO mixture was incubated for 1 h at 37 °C, and thereafter irradiated with long wave UV from a TLC lamp (365 nm) for 1 h. The irradiated fibrils were then boiled in SDS (25 mM final concentration), and subjected to $CHCl_3$/MeOH precipitation to remove excess detergent and buffer as previously described.[2] The translucent brown protein pellet was then resuspended in 40 μL of sterilized, Milli-Q water. At this point, the whole protein sample was subjected to MALDI-MS analuysis by spotting 1 μL of sample in 1μL of sinapic acid matrix, prepared as a saturated solution in a 50:50 mixture of ACN:H_2O with 0.1% TFA. The remaining sample was digested using trypsin (Promega Corporation) at 37 °C overnight (50 mM NH_4CO_3, 1 mM $CaCl_2$, 1:50-1:100 protein:trypsin ratio). The resulting tryptic peptide mixture was diluted 1:10 in sterile water with 0.1% TFA, and analyzed via MALDI-MS by spotting 1 μL of sample in 1 μL of CHCA matrix or DHB matrix, prepared as saturated solutions in a 50:50 mixture of ACN:H_2O with 0.1% TFA. Crosslinked peptide matching was accomplished by plotting a matrix of theoretical peptide fragments or theoretical peptide + **ClX1** or + **ClX2** vs. those acquired experimentally from the MALDI-MS. By subtracting the masses from each other, matches within 1.0 Da were identified. A fragment corresponding to residues 81-96, a component of site 9, was observed crosslinked in **ClX1** samples (Fig. S6). A fragment corresponding to residues 22-34, a component of site 2, was observed crosslinked in **ClX2** samples (Fig. S7). This was the only mass found to be present in the samples with **ClX2** and absent in the DMSO control samples.

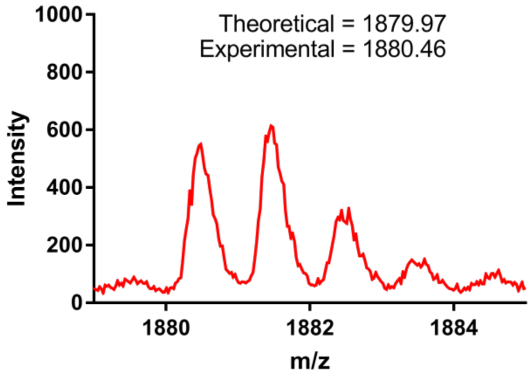

Peptide Fragment	Missed Cleavages	WT αS (m/z)	+ ClX1 (m/z)
1-6	0	770.36	1171.54
1-10	1	1155.59	1556.78
1-12	2	1354.72	1755.91
1-21	3	2209.17	2610.36
7-10	0	404.25	805.44
7-12	1	603.38	1004.57
7-21	2	1457.83	1859.02
7-23	3	1686.97	2088.16
11-12	0	218.15	619.34
11-21	1	1072.60	1473.78
11-23	2	1301.74	1702.93
11-32	3	2113.16	2514.35
13-21	0	873.47	1274.65
13-23	1	1102.61	1503.80
13-32	2	1914.03	2315.21
13-34	3	2143.17	2544.36
22-23	0	248.16	649.35
22-32	1	1059.58	1460.76
22-34	2	1288.72	1689.91
22-43	3	2221.22	2622.40
24-32	0	830.44	1231.62
24-34	1	1059.58	1460.76
24-43	2	1992.08	2393.26
24-45	3	2221.22	2622.40
33-34	0	248.16	649.35
33-43	1	1180.66	1581.84
33-45	2	1409.80	1810.99
33-58	3	2686.48	3087.66
35-43	0	951.51	1352.70
35-45	1	1180.66	1581.84
35-58	2	2457.33	2858.52
35-60	3	2686.48	3087.66
44-45	0	248.16	649.35
44-58	1	1524.84	1926.02
44-60	2	1753.98	2155.17
44-80	3	3663.01	4064.19
46-58	0	1295.70	1696.88
46-60	1	1524.84	1926.02
46-80	2	3433.86	3835.05
59-60	0	248.16	649.35
59-80	1	2157.19	2558.37
59-96	2	3616.95	4018.14
59-97	3	3745.05	4146.23
61-80	0	1928.04	2329.23
61-96	1	3387.81	3789.00
61-97	2	3515.91	3917.09
81-96	0	1478.78	**1879.97**
81-97	1	1606.88	2008.07
81-102	2	2148.17	2549.35
97-102	1	688.40	1089.58
98-102	0	560.30	961.49

Fig. S6 Photo-crosslinking of **ClX1** to αS Fibrils. Top: Tryptic digest MALDI-MS of αS fibrils irradiated with **ClX1** showing m/z corresponding to crosslinked 22-34 fragment. Bottom: List of $(M+H)^+$ masses of theoretical tryptic fragments of unmodified wild-type αS (WT αS) or αS crosslinked to **ClX1** (+ ClX2). Green: Fragments observed, as expected, in only the DMSO experiment or the **ClX1** experiment. Bolded mass corresponds to crosslinked 81-96 fragment. Yellow: Observed in both DMSO and **ClX1** experiments. Red: Not observed in either experiment. Blue: Observed in other condition. Grey: Ambiguous, isobaric mass.

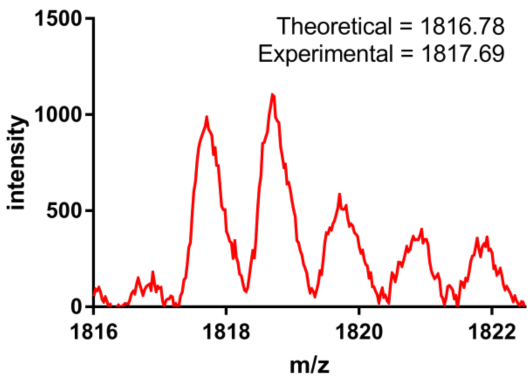

8

Peptide Fragment	Missed Cleavages	WT αS (m/z)	+ ClX2 (m/z)
1-6	0	770.36	1298.41
1-10	1	1155.59	1683.64
1-12	2	1354.72	1882.78
1-21	3	2209.17	2737.23
7-10	0	404.25	932.31
7-12	1	603.38	1131.44
7-21	2	1457.83	1985.89
7-23	3	1686.97	2215.03
11-12	0	218.15	746.20
11-21	1	1072.60	1600.65
11-23	2	1301.74	1829.80
11-32	3	2113.16	2641.22
13-21	0	873.47	1401.52
13-23	1	1102.61	1630.66
13-32	2	1914.03	2442.08
13-34	3	2143.17	2671.23
22-23	0	248.16	776.22
22-32	1	1059.58	1587.63
22-34	2	1288.72	**1816.78**
22-43	3	2221.22	2749.27
24-32	0	830.44	1358.49
24-34	1	1059.58	1587.63
24-43	2	1992.08	2520.13
24-45	3	2221.22	2749.27
33-34	0	248.16	776.22
33-43	1	1180.66	1708.71

Peptide Fragment	Missed Cleavages	WT αS (m/z)	+ ClX2 (m/z)
33-45	2	1409.80	1937.85
33-58	3	2686.48	3214.53
35-43	0	951.51	1479.57
35-45	1	1180.66	1708.71
35-58	2	2457.33	2985.39
35-60	3	2686.48	3214.53
44-45	0	248.16	776.22
44-58	1	1524.84	2052.89
44-60	2	1753.98	2282.04
44-80	3	3663.01	4191.06
46-58	0	1295.70	1823.75
46-60	1	1524.84	2052.89
46-80	2	3433.86	3961.92
59-60	0	248.16	776.22
59-80	1	2157.19	2685.24
59-96	2	3616.95	4145.01
59-97	3	3745.05	4273.10
61-80	0	1928.04	2456.10
61-96	1	3387.81	3915.87
61-97	2	3515.91	4043.96
81-96	0	1478.78	2006.84
81-97	1	1606.88	2134.93
81-102	2	2148.17	2676.22
97-102	1	688.40	1216.45
98-102	0	560.30	1088.36

Fig. S7 Photo-crosslinking of **ClX2** to αS Fibrils. Top: Tryptic digest MALDI-MS of αS fibrils irradiated with **ClX2** showing m/z corresponding to crosslinked 22-34 fragment. Bottom: List of $(M+H)^+$ masses of theoretical tryptic fragments of unmodified wild-type αS (WT αS) or αS crosslinked to **ClX2** (+ ClX2). Green: Fragments observed, as expected, in only the DMSO experiment or the **ClX2** experiment. Bolded mass corresponds to crosslinked 22-34 fragment. Yellow: Observed in both DMSO and **ClX2** experiments. Red: Not observed in either experiment.

***In Vitro* Radioligand Binding Assays**

Screening compound library. Compounds **1-17** (Fig. S8) were purchased from vendors that were listed on the ZINC15 compound library.[3] Compounds were screened for αS binding and after identifying the lead compound, **6**, the core structure of compound **6** and its pyrazole or oxadiazole derivatives were used for similarity search on the Mcule, Inc. website.[12] The similarity threshold was set to higher than 0.8, then compound **18-56** (Fig. S9) were chosen and ordered from the four companies mentioned above.

In order to screen the purchased compounds for αS binding, 100 nM solutions of each compound (**1-56**) were incubated for 1 hour at 37 °C with 100 nM αS fibrils and [^{3}H]**Tg-190b** (6 nM) or [^{3}H]**BF2846** (3 nM) in 50 mM Tris-HCl, pH 7.4. Total binding was measured in the absence of competitor and nonspecific binding was determined in reactions containing unlabeled **Tg-190b** (1 µM) or **BF2846** (0.5 µM). After incubation, bound and free radioligand were separated by vacuum filtration through Whatman GF/C filters in a 24-sample harvester system (Brandel; Gaithersburg, MD, USA), followed by washing with buffer containing 10 mM Tris-HCl (pH 7.4) and 150 mM NaCl. Filters containing the bound ligand were mixed with 3 mL of scintillation cocktail (MicroScint-20, PerkinElmer; Waltham, MA, USA) and counted after 12 h of incubation on a MicroBeta System (PerkinElmer). All data points were acquired in triplicate. Percentage of bound radioligand relative to total binding was plotted and data was analyzed by One-Way ANOVA, comparing the mean of each data set to the mean of total binding. These data are shown in Fig. 2 in the main text, and Fig. S8 and S10.

a

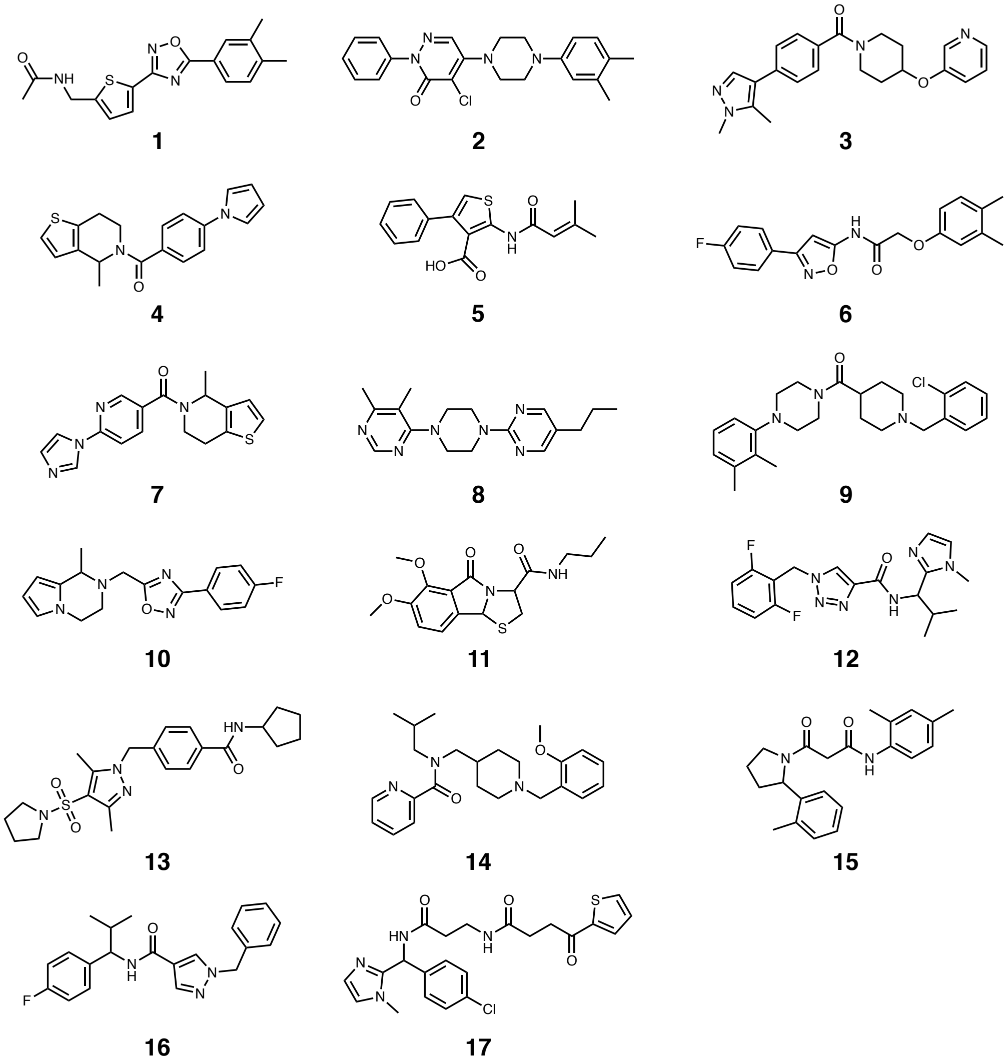

b

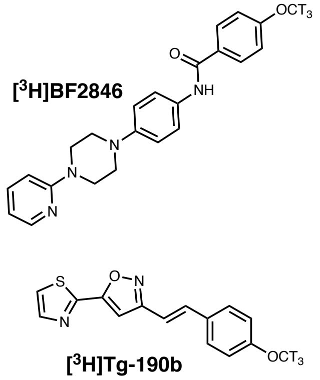

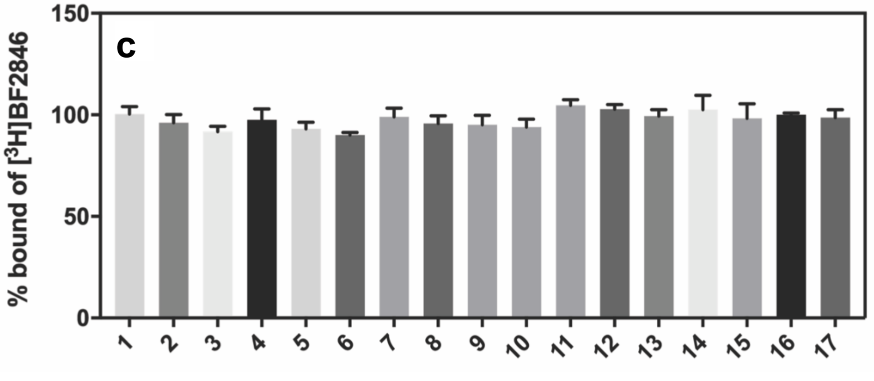

Fig. S8 Initial Screening of Compounds from Exemplar Search. a. Molecular structures of 17 compounds from the Exemplar screening. Compound **6** showed high affinity for αS fibrils *in vitro* and its core structure was used for similarity search. b. Molecular structures of radioligands used in the screening and competition binding assays. c. Site 9 radioligand competition assays with $[^3H]$**BF2846**. $[^3H]$**Tg-190b** competition binding data are shown in Fig. 2a of the main text.

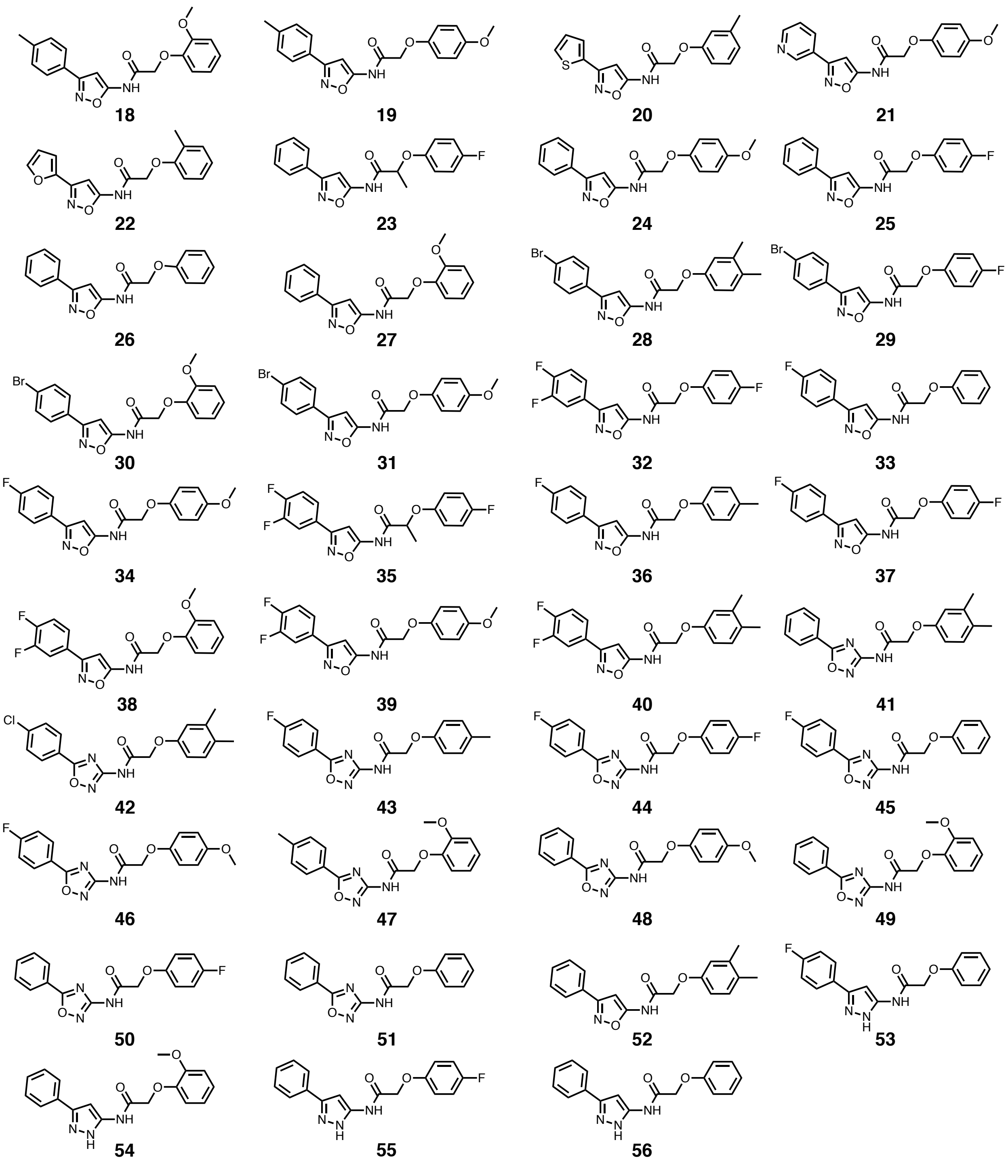

Fig. S9 Compounds Used in Structure Activity Relationship Study. Competition binding studies were performed with these 39 compounds from the similarity search.

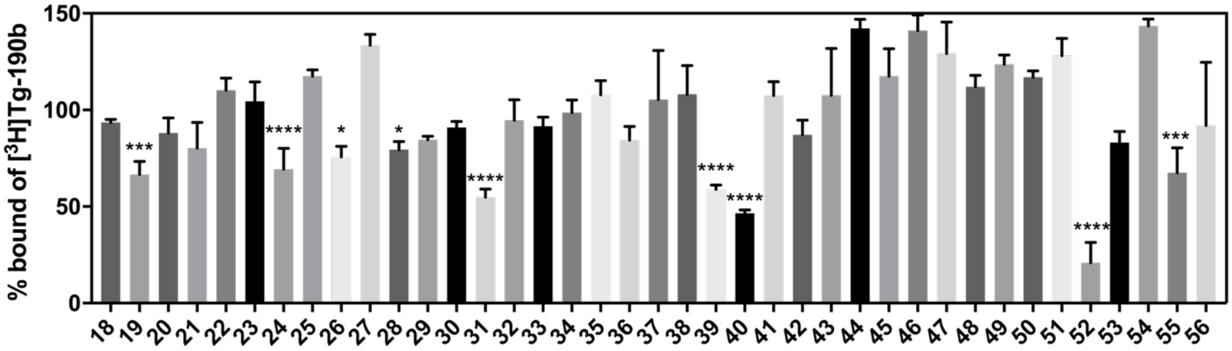

Fig. S10 Screening of SAR Library Derived from Compound **6**. Site 2 radioligand competition binding assays with [^{3}H]**Tg-190b**. ANOVA was performed with Dunnett's multiple comparisons test, where *P<0.0332, **P<0.0021, ***P<0.0002 and ****P<0.0001. A subset of these data is shown in Fig. 3 of the main text.

Competition binding assay. αS fibrils (100 nM for Site 2 and 50 nM for Site 9) were mixed with Site 2 ligand [^{3}H]**Tg-190b** (6 nM) or Site 9 ligand [^{3}H]**BF2846** (3 nM) and varying concentrations of unlabeled compounds **2**, **6**, **24**, **28**, **31**, **39**, **40**, or **52**. Compounds were diluted in 50 mM Tris-HCl buffer (pH 7.4) and mixed with fibrils and radioligand in a total volume of 150 µL. Total binding was measured in the absence of competitor and nonspecific binding was determined in reactions containing unlabeled **Tg-190b** (1 µM) or **BF2846** (0.5 µM). In a duplicate set of binding reaction, fibrils were replaced with equal volume of buffer to measure the amount of radioligand binding to the filter paper. Reactions were incubated at 37 °C for 1 h. After incubation, bound and free radioligand were separated by vacuum filtration through Whatman GF/C filters (Brandel) in a 24-sample harvester system (Brandel), followed by washing with buffer containing 10 mM Tris-HCl (pH 7.4) and 150 mM NaCl. Filters containing the bound ligand were mixed with 3 mL of scintillation cocktail (MicroScint-20, PerkinElmer) and counted after 12 h of incubation on a MicroBeta System (PerkinElmer). All data points were acquired in triplicate.

The measured cpm values were converted to % specific binding as described by the equation below:

$$\% \text{ specific binding} = \frac{binding\ in\ the\ presence\ of\ competitor - nonspecific\ binding}{total\ binding - nonspecific\ binding} * 100$$

IC_{50} values were calculated by fitting the data to Equation S1 below by non-linear regression, using GraphPad Prism software:

$$Y = \text{Bottom} + (\text{Top-Bottom}) / (1 + 10^{(X - logIC_{50})}) \qquad \text{(S1)}$$

where logIC_{50} is the log of the concentration of competitor that results in binding half-way between Bottom and Top; Top and Bottom are plateaus in the units of Y axis; they are binding observed in the absence of competitor and in the presence of a maximal concentration of competitor, respectively. Binding curves for compounds **2** and **6** are shown in Fig. 2 in the main text and for compounds **6**, **24**, **28**, **31**, **39**, **40**, and **52** in Figs. S11 and S12. Their IC_{50} values are given in Table 1 in the main text.

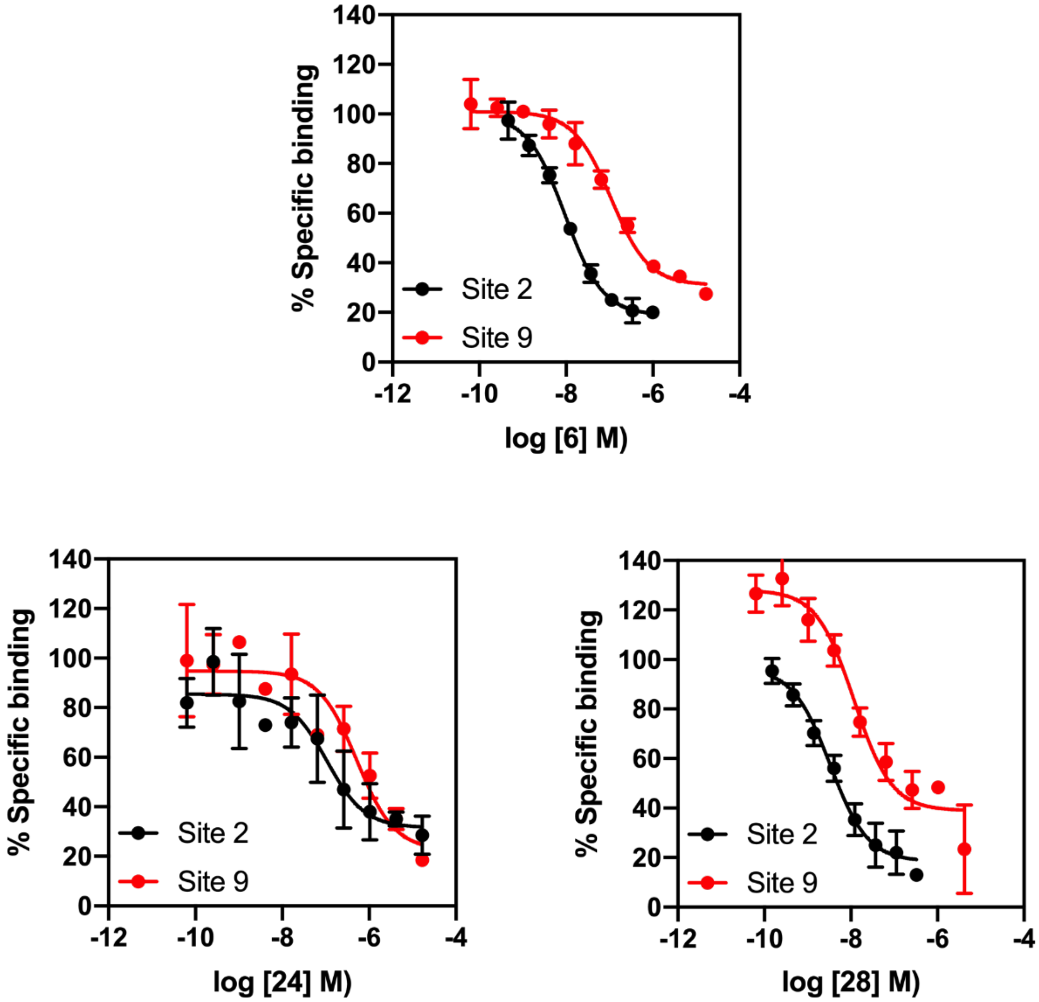

Fig. S11 Competition binding curves for αS fibrils (100 nM) with compounds **6, 24**, and **28**. αS fibrils were incubated with [^{3}H]**Tg-190b** (Site 2) or [^{3}H]**BF2846** (Site 9) and increasing concentrations of competitors (**6, 24**, or **28**). Data points represent mean ± s.d. (n=3).

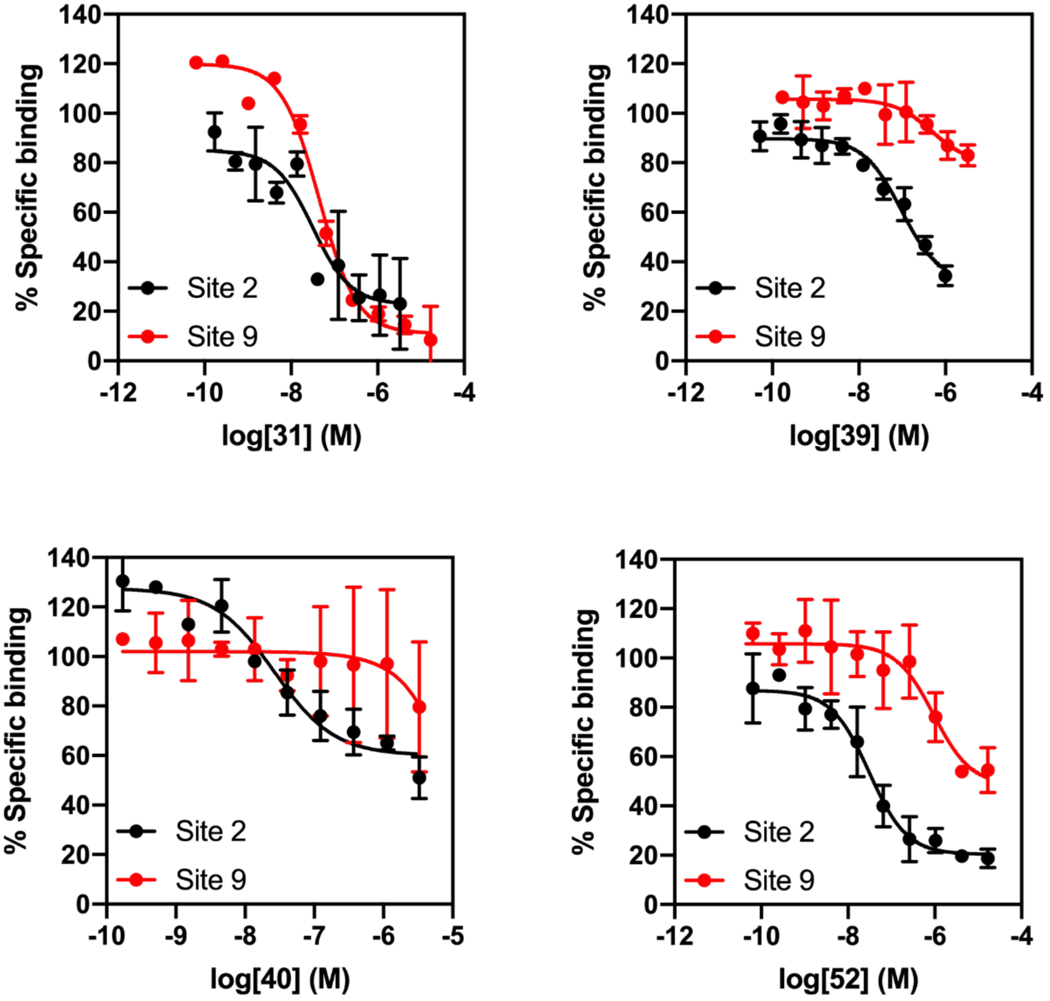

Fig. S12 Competition binding curves for αS fibrils (100 nM) with compounds **31**, **39**, **40**, and **52**. αS fibrils were incubated with [^{3}H]**Tg-190b** (Site 2) or [^{3}H]**BF2846** (Site 9) and increasing concentrations of competitors (**31**, **39**, **40**, or **52**). Data points represent mean ± s.d. (n=3).

Saturation binding assay. αS (50 nM) or Aβ42 (100 nM) fibrils were incubated for 1 h at 37 °C with increasing concentrations of [^{125}I]**61** in 50 mM Tris-HCl, pH 7.4, in a total volume of 150 μL. Nonspecific binding was determined in a duplicate set of binding reactions containing 2 μM unlabeled **61**. To measure the amount of radioligand binding to the filter paper, fibrils were replaced with equal volume of buffer in a duplicate set of binding reactions. After incubation, bound and free radioligand were separated by vacuum filtration through Whatman GF/C filters (Brandel) in a 24-sample harvester system (Brandel), followed by washing with buffer containing 10 mM Tris-HCl (pH 7.4) and 150 mM NaCl. Filters containing the bound ligand were counted immediately on 2470 WIZARD Automatic Gamma Counter (Perkin Elmer). All data points were acquired in triplicate. The equilibrium dissociation constant (K_d) and the maximal number of binding sites (B_{max}) were determined by globally fitting the total binding and nonspecific binding data to Equation S2:

$$Y = B_{max}*[X/(K_d+X)] + NS*X \qquad (S2)$$

where X is the concentration of ligand ([^{125}I]**61**) and NS is nonspecific binding, using GraphPad Prism software (San Diego, CA, USA).

Photo-crosslinking in Mouse Brain Lysate

Total Mouse Brain Protein Preparation. Mice were anesthetized using isoflurane gas in a closed chamber (AKORN, Lake Forest, IL, USA) followed by cervical dislocation. Brains were quickly dissected and snap-frozen in dry ice. Mouse brains were mechanically homogenized in five-fold excess (w/w) RIPA buffer (Thermo Fisher Scientific; Waltham, MA, USA) using a ground glass tissue plunger. Homogenized sample was then subjected to probe sonication (Qsonica, Newtown, CT, USA) on ice using a microtip, at an amplitude of 50% at intervals of 10 s on 20 s off, for a total process-time (on-time) of 5 minutes. Crude lysate was then clarified by centrifugation at 10,000xg at 4 °C for 60 min. The clarified lysate was then spin-concentrated to approximately 1 ml using Amicon 3 kDa molecular weight cutoff filters (MilliporeSigma). Residual precipitate was removed via an additional centrifugation step at 10,000xg for 60 min. The lysate total protein concentration was then determined using the Bio-Rad detergent compatible (DC) protein assay (Hercules, CA, USA).

Photo-crosslinking and Click Chemistry. Samples and controls were prepared by doping 50 µL of PBS or *in vitro* prepared αS fibrils (50 µM final concentration) into 50 µL of mouse brain lysate (10 mg/ml final concentration), followed by **CLX2** in DMSO (50 µM final concentration) or DMSO vehicle control in 2 ml glass vials. Controls containing αS were prepared by diluting 50 µL of *in vitro* fibrils (100 µM) with 50 µL of PBS, for a final concentration of 50 µM. Samples were then allowed to incubate for 1-2 h at 37 °C. Following incubation, samples were irradiated with 365 nm light using a TLC lamp for 30 min. Fibrils were labelled using azide-alkyne cycloaddition (click) chemistry, with BODIPY-FL azide. (Lumiprobe, Hunt Valley, MD, USA) Briefly, a "click-mix" was prepared by mixing 10 mM copper sulfate and 50 mM THPTA in a 5:2 ratio (i.e. 20 µL of copper sulfate and 8.0 µL of THPTA). BODIPY-FL azide was then added to

samples (50 µM final concentration), followed by "click-mix" (50 µM copper sulfate, 100 µM THPTA final concentration), sodium ascorbate (200 µM final concentration), and PBS to achieve a final volume of 200 µL. Reaction was allowed to go overnight at room temperature with shaking (500 rpm). Samples were then boiled in SDS (50 mM final concentration) for 60 min, and allowed to cool to room temperature. Samples were then analyzed via SDS-PAGE using a 4-12% Bis-Tris pre-cast gel (NuPAGE, Thermo Fisher Scientific). αS monomer labeled with BODIPY-FL maleimide (prepared as previously described[13]) was used as a control. Residual binding of the BODIPY fluorophore was noted in αS lanes in the absence of **CLX2**; however, this was lower than in lanes dosed with the **CLX2** probe. Comparison of lanes 4 and 5 in Fig. S13 shows that αS is labeled by crosslinking in the presence of mouse brain lysate proteins.

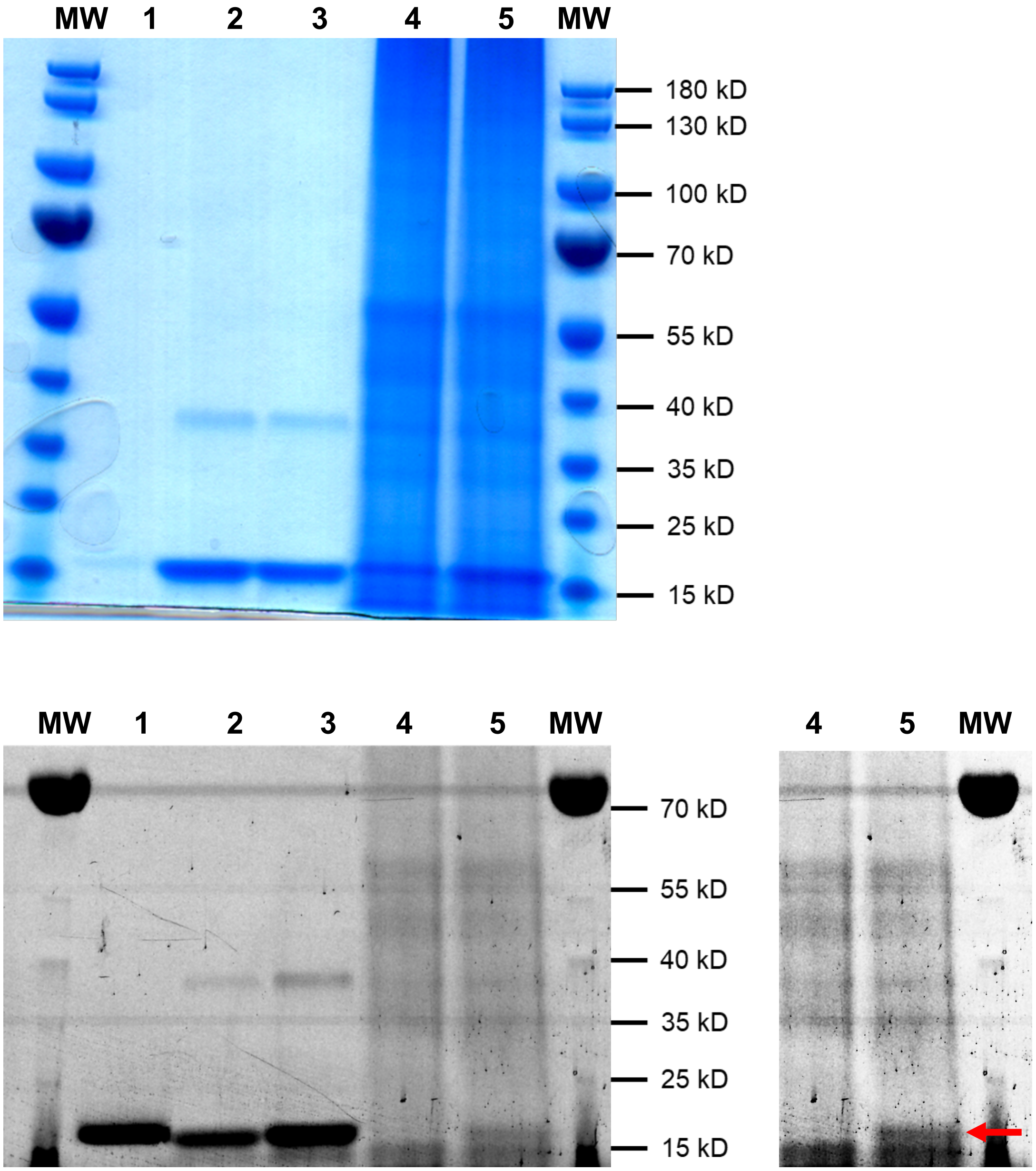

Fig. S13 PAGE Analysis of **CLX2** Crosslinking in Mouse Brain Lysate. Crosslinking followed by fluorescent labeling through click chemistry shows labeling of αS fibrils in the presence of mouse brain lysate proteins. Top: Coomassie staining of total protein. Bottom Left: Fluorescence imaging of BODIPY FL probe. Bottom Right: Gel fluorescence with increased contrast for better visualization of brain lysate lanes. Lanes: Molecular weight markers (MW), BODIPY Fl labeled αS (1), αS fibrils (2), αS fibrils + **CLX2** (3), lysate + αS fibrils (4), lysate + αS fibrils + **CLX2** (5). Red arrow indicates position of αS.

Mouse Brain Radioligand Binding Assays

Animals. A53T (B6C3-Tg(Prnp-SNCA*A53T)83Vle/J) and B6C3F1/J mice were obtained from The Jackson Laboratory (Bar Harbor, ME, USA). All animal studies were performed under protocols approved by the University of Pennsylvania Institutional Animal Care and Use Committee. Animals were euthanized by cervical dislocation under isoflurane anesthesia at 15 or 17 months of age and the brain was extracted for autoradiography and microscopy.

***In vitro* autoradiography**. Blocks of mouse brain tissue (17-month old) were frozen in optimal cutting temperature (OCT) compound. The frozen tissue was sliced into 10 μM thick sections in a Leica CM1950 cryostat and mounted onto Apex Superior Adhesive slides (Leica; Buffalo Grove, IL, USA). Frozen sections of both A53T and B6C3F1/J mouse brain tissue were thawed at room temperature for 20 min, then washed with 40% ethanol in DPBS (Gibco) for 5 min. Next, sections were incubated (1 hour at room temperature) with 40% ethanol in DPBS containing either [^{125}I]**61** (6 nM) alone or [^{125}I]**61** (6 nM) with 20 μM unlabeled **Tg-190b**. After incubation, sections were washed in ice-cold 40% ethanol in DPBS (2x30 s), followed by a wash in ice-cold Milli-Q water (1 min). Sections were dried in a stream of air, exposed to a phosphor screen (GE Healthcare; Chicago, IL, USA) and the screen was imaged on a Typhoon FLA 7000 phosphor imager (GE Healthcare; Chicago, IL, USA). Raw autoradiography images were imported to MATLAB R2017b (MathWorks Inc.; Natick, MA, USA) to extract individual images. Each autoradiography image was manually registered to the corresponding staining image by using PMOD image analysis software (PMOD Technologies Ltd; Zurich, Switzerland). Example data are shown in Fig. S14.

Immunofluorescence. Frozen brain tissue sections, adjacent to the ones used for autoradiography, were thawed at room temperature for 20 min. Sections were fixed with 4% paraformaldehyde in

PBS, washed with PBS three-times, then permeabilized with 0.1% Triton X-100 in PBS. Sections were blocked with 10% normal goat serum (Fisher Scientific) at room temperature for 1 hour, then with goat F(ab) anti-mouse IgG H&L (1:1000 in 1% normal goat serum in PBS with 0.2% Tween-20; abcam, ab6668) for 1 hour at RT. After blocking, sections were incubated with primary anti-αS (phosphor S129) antibody (81A; 1:1000 in 1% normal goat serum in PBST) overnight at 4 °C. The 81A antibody was provided by the laboratory of Virginia Lee.[13, 14] After three washes with PBST, the tissue was incubated with a secondary antibody labeled with Alexa Fluor 488 (1:500 in 1% normal goat serum in PBST; Invitrogen) for 1 hour at room temperature. Tissue was washed with PBST twice, then with PBS and sealed under a coverslip. The fluorescent images were acquired by a Zeiss Axio Imager M2 microscope (Oberkochen, Germany). Example data are shown in Fig. S14.

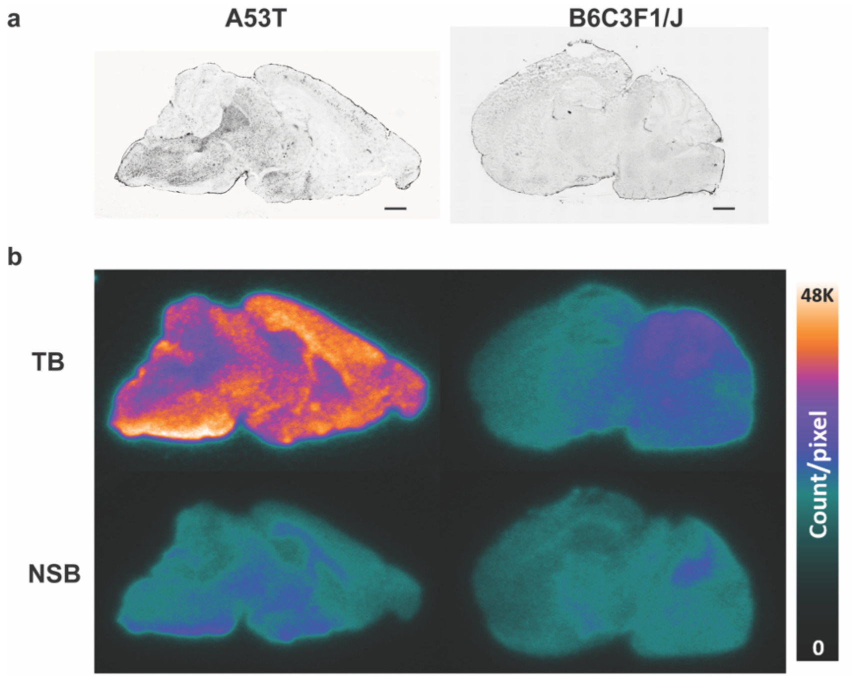

Fig. S14 *In vitro* Autoradiography on Mouse Brain Tissue Sections to Assess [^{125}I]**61** Binding. a. Immunofluorescence staining of A53T and normal (B6C3F1/J) mouse brain sections with PS129 anti-αS antibody (scale bar: 1000 μm). b. Autoradiograms showing the binding of [^{125}I]**61** in A53T and normal mouse brain sections. The upper two sections are total binding (TB) and the lower two sections are non-specific binding (NSB), defined using non-labeled **Tg-190b** (20 μM).

Preparation of sarkosyl-insoluble fraction from disease mouse brain. Sarkosyl-insoluble fractions were prepared as previously described.[15] In brief, A53T mouse brain (15-month old) was homogenized in high-salt (HS) buffer (50 mM Tris-HCl pH 7.4, 750 mM NaCl, 10 mM NaF, 5 mM EDTA) with protease and protein phosphatase inhibitors, incubated on ice for 20 min and centrifuged at 100,000xg for 30 min. The pellet was then re-extracted with HS buffer, followed by sequential extractions with 1% Triton X-100-containing HS buffer and 1% Triton X-100-containing HS buffer with 30% sucrose. The pellet was then resuspended in 1% sarkosyl-containing HS buffer, incubated at 4 °C overnight on a rotisserie and centrifuged at 100,000xg for 30 min. The resulting sarkosyl-insoluble pellet was washed once with DPBS and resuspended in DPBS by brief sonication (20 pulses, 1 s per pulse). This suspension was termed the 'sarkosyl-insoluble fraction' which contained pathological αS. The amount of αS in the fraction was determined by sandwich ELISA (kit from ThermoFisher Scientific; catalog # KHB0061) following manufacturer's protocol.

***In vitro* autoradiography on sarkosyl-insoluble fraction**. The sarkosyl-insoluble fraction was frozen in OCT (Tissue-Tek, Sakura Finetek; Torrance, CA, USA). The frozen fraction was sliced into 10 µM thick sections in a Leica CM1950 cryostat and mounted onto Apex Superior Adhesive slides (Leica). Frozen sections were thawed at room temperature for 20 min, then washed with 40% ethanol in PBS for 5 min. Next, sections were incubated (1 h at room temperature) with 40% ethanol in PBS containing either [^{125}I]**61** (1 nM) alone or [^{125}I]**61** (1 nM) with 100 nM unlabeled **61**. After incubation, sections were washed in ice-cold 40% ethanol in PBS (3x1 min), followed by a wash in ice-cold Milli-Q water (1 min). Sections were dried in a stream of air, exposed to a storage phosphor screen (GE Healthcare) and the screen was imaged on a Typhoon FLA 7000 phosphor imager. Autoradiography images were quantified using Fujifilm Multi Gauge software.

Computational Docking and Comparison of Fibril Structures

Molecular docking using Autodock. *In silico* molecular docking studies were performed following previously published protocol.[2] All the compound structures were drawn in ChemDraw Profession 15.1 (PerkinElmer Informatics, Inc.), then imported to Chem3D Ultra 15.1 (PerkinElmer Informatics, Inc.) to minimize individual structures by MMFF94 force field for preparation of molecular docking. Molecular blind docking studies were performed via the AutoDock 4.2 plugin on PyMOL (pymol.org).[16] The solid-state NMR structure of full-length αS fibril (2N0A) was used as a target protein for blind docking. Non-polar hydrogens were removed from both protein and compound structures. A grid box with a dimension of 95 × 50 × 95 $Å^3$ was applied to αS fibril structure. The Lamarckian Genetic Algorithm with a maximum of 2,500,000 energy evaluations was used to calculate 1,000 protein-ligand binding poses for each compound. The % probability as well as the average and best binding energy (BE) of each binding site determined from blind docking are reported in Table S1.

Table S1. Molecular docking of compounds **6, 24, 28, 31, 39, 40** and **52** to Site 2 in αS fibrils (PDB 2N0A).

**6**	Probability (%)	7.80%
	Average BE	-5.84±0.38
	Best BE	-6.56
**24**	Probability (%)	15.20%
	Average BE	-5.58±0.39
	Best BE	-6.24
**28**	Probability (%)	14.30%
	Average BE	-6.67±0.42
	Best BE	-7.42
**31**	Probability (%)	13.80%
	Average BE	-6.26±0.39
	Best BE	-6.96
**39**	Probability (%)	8.20%
	Average BE	-5.55±0.37
	Best BE	-6.30
**40**	Probability (%)	13.10%
	Average BE	-5.89±0.36
	Best BE	-6.85
**52**	Probability (%)	14.20%
	Average BE	-6.09±0.39
	Best BE	-7.05

Molecular docking through Exemplar alignment. Compounds were docked and minimized into six unique fibril architectures (2N0A, 6A6B, 6CU7, 6XYO, 6XYP and 6XYQ) that represented possible folds of Site 2.[1, 17-19] Sites were identified in all alternative structures by generating exemplars for each structure of residues 38 – 46 which correspond to Site 2 in the 2N0A structure. Compound **6** was aligned to the Site 2 exemplar using the following command in Shape-It from Silicos-It:[20]

" shape-it -r reference_exemplar.pdb -d compound6.pdb -o compound6_aligned.pdb -s scores.txt –noRef "

Additional derivatives were then aligned to the parent compound directly also using the following command in AlignIt from SilicosIt:

"align-it -r reference_mol.pdb –refType ent -d db_mol.pdb –dbType ent -p db_mol.pharm -o db_mol_aligned.pdb –outType ent -s scores.txt "

Following molecular alignment of the compounds to their respective exemplars, complexes were minimized through MinMover in PyRosetta. Minimization was performed using the beta_nov16_cart score function with the lbfgs_strong_wolfe optimizer, 2000 maximum steps and a value of 0.000001 for the tolerance.[21] During minimization, sidechain movements were allowed for residues 38 – 46, which comprise the Site 2 pocket, while backbone sampling of the fibril was not performed. The total ligand score (TLS) was computed for each molecule in the minimized complex and is the linear combination of weighted score terms in the beta_nov16 score function that is attributable to the ligand and any interactions it makes with the protein.

Correlation of ΔΔG values. Linear regressions of ΔΔG values from molecular docking with ΔΔG values derived from experimental IC_{50} measurements were used to determine whether the Autodock BE values or PyRosetta TLS values were more correlative. TLS values from PyRosetta were converted into ΔΔG values using Equation S3. *TLS(a)* and *TLS(b)* represent the total ligand score computed for any pair of molecules a and b. Additionally, α represents the scaling factor used to convert between Rosetta energy units (REU) and units of kcal/mol. Here, α was set to 1/2.94 as this value was previously determined from benchmarking of the closely related ref2015 score function,[22] which has undergone minor optimization to generate the beta_nov16 score function which has yet to be benchmarked.

$$\Delta\Delta G_{TLS} = \alpha(TLS(a) - TLS(b)) \qquad \text{(S3)}$$

Similarly, we can compute ΔΔG values from the average and maximum BE values determined from Autodock simulations using Equation S4.

$$\Delta\Delta G_{BE} = BE(a) - BE(b) \qquad \text{(S4)}$$

Experimental ΔΔG values were computed using Equation S5 where the ΔΔG for a pair of molecules a and b can be determined from the ratio of their IC_{50} values.

$$\Delta\Delta G_{Exp} = -RTln\left(\frac{IC_{50}(a)}{IC_{50}(b)}\right) \qquad \text{(S5)}$$

Correlations between the experimental and simulated ΔΔG values can be seen in Fig. 7 in the main text for TLS-derived values and in Fig. S15 below for values derived from Autodock BE.

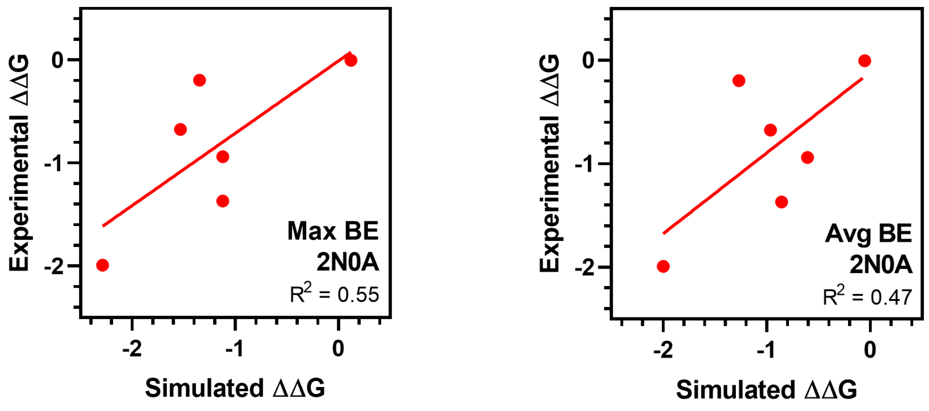

Figure S15: Simulated ΔΔG versus experimental ΔΔG for Autodock simulations. Simulated ΔΔG were computed using either the best (left) or average (right) binding energy values reported in Table S1.

Comparison of fibril structures. We found that simulated values from PyRosetta provided a significantly better correlation with the experimental ΔΔG values than the values computed from Autodock. Therefore, PyRosetta TLS values were used for comparing 2N0A with other fibril structures. In spite of variable overall fibril folds and protofibril packing, the Site 2 regions (residues 38-46) of all of the fibril structures can be aligned reasonably well (Figs. S16 and S17). We compared this region to the electron density map used to generate the 6XYQ structure and found that several structures aligned well. The high levels of experimental correlation observed for TLS values calculated using the 6XYQ and 6XYP Site 2 regions indicate that these can also provide good models for analyzing SAR data.

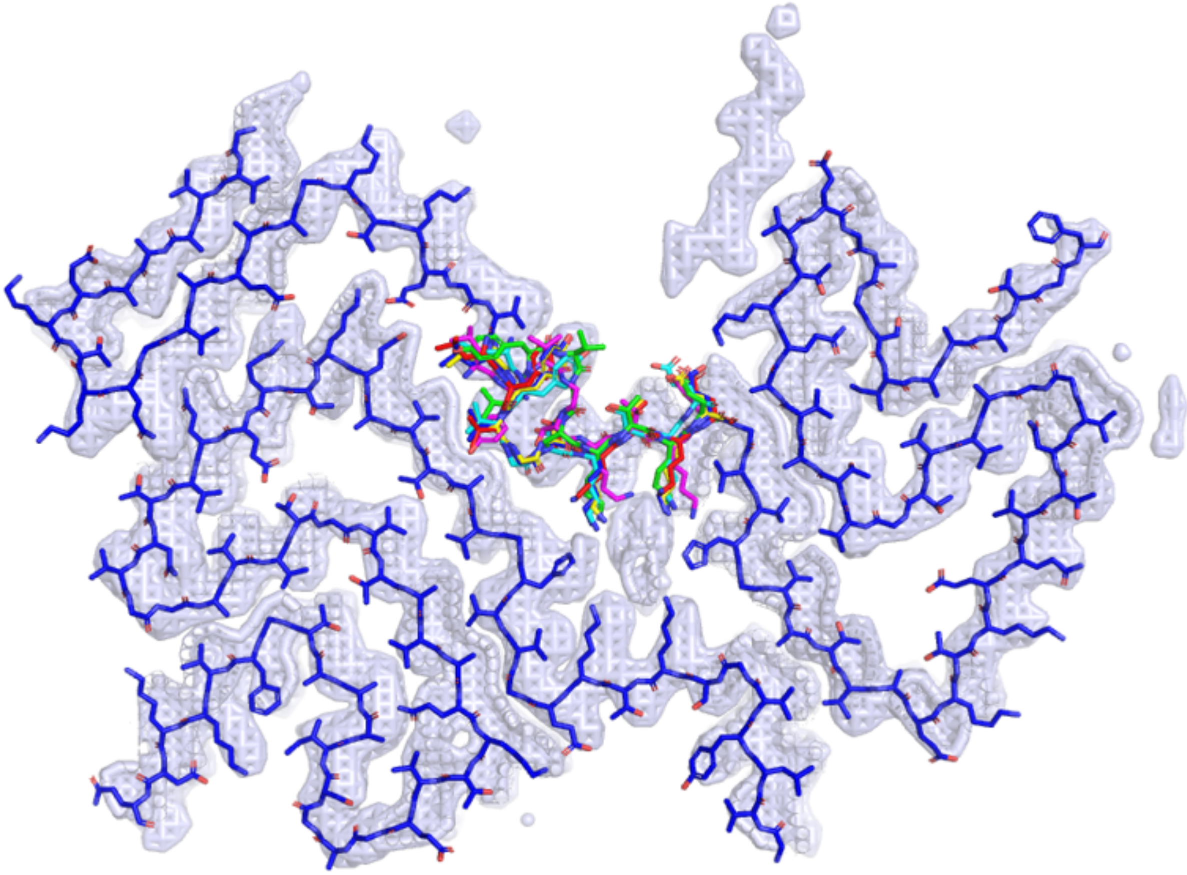

Fig. S16: Comparison of structural data from different fibril forms. Structure of 6XYQ (blue, sticks) along with the experimental electron density used to refine the 6XYQ structure (light-blue, mesh) and alternative fibrils structures (2N0A: red, sticks; 6XYP: yellow, sticks; 6A6B: magenta, sticks; 6XYO: cyan, sticks; and 6CU7: green, sticks).

Fig. S17 (following page): Comparison of structural data with simulated ΔΔG versus experimental ΔΔG for PyRosetta simulations. Zoom in of residues 38-46 for each structure rendered within the 6XYQ electron density (right) alongside the correlation between the simulated ΔΔG from PyRosetta and the calculated experimental ΔΔG.

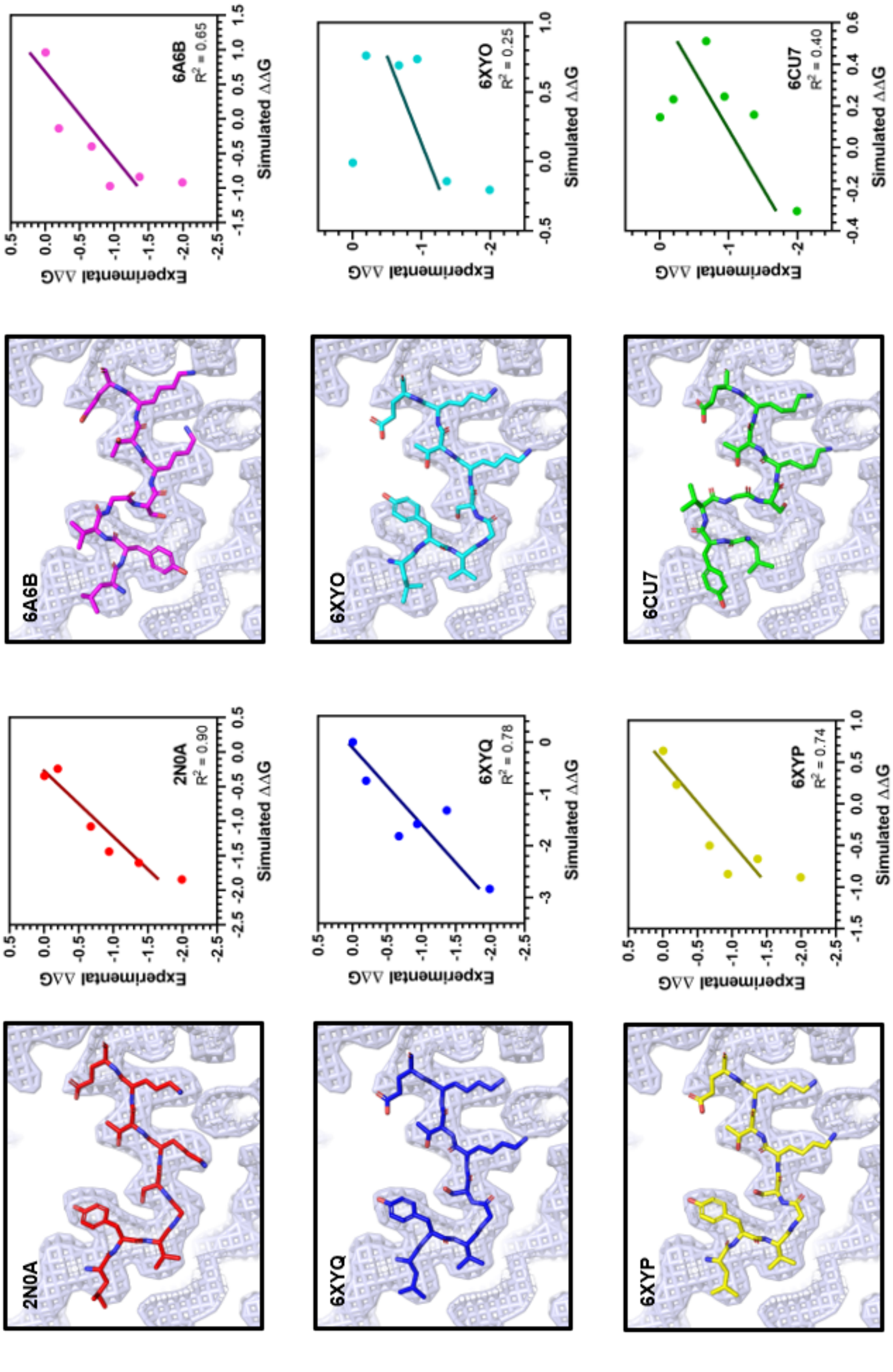
2N0A
6XYQ
6XYP
6A6B
6XYO
6CU7
2N0A
R2 = 0.90
6XYQ
R2 = 0.78
6XYP
R2 = 0.74
6A6B
R2 = 0.65
6XYO
R2 = 0.25
6CU7
R2 = 0.40
Experimental ΔΔG
Simulated ΔΔG

References

1. M. D. Tuttle, G. Comellas, A. J. Nieuwkoop, D. J. Covell, D. A. Berthold, K. D. Kloepper, J. M. Courtney, J. K. Kim, A. M. Barclay, A. Kendall, W. Wan, G. Stubbs, C. D. Schwieters, V. M. Y. Lee, J. M. George and C. M. Rienstra, *Nat. Struct. Mol. Biol.*, 2016, **23**, 409.
2. C.-J. Hsieh, J. J. Ferrie, K. Xu, I. Lee, T. J. A. Graham, Z. Tu, J. Yu, D. Dhavale, P. Kotzbauer, E. J. Petersson and R. H. Mach, *ACS Chem. Neurosci.*, 2018, **9**, 2521.
3. T. Sterling and J. J. Irwin, *J. Chem. Inf. Model.*, 2015, **55**, 2324.
4. J. Taminau, G. Thijs and H. De Winter, *J. Mol. Graph. Model.*, 2008, **27**, 161.
5. S. W. Reilly and R. H. Mach, *Organic Letters*, 2016, **18**, 5272.
6. Y. Wang, Z. Fang, X. Chen and Y. Wang, *Organic Letters*, 2018, **20**, 5732.
7. Z. Lengyel-Zhand, J. J. Ferrie, B. Janssen, C.-J. Hsieh, T. Graham, K.-Y. Xu, C. M. Haney, V. M. Y. Lee, J. Q. Trojanowski, E. J. Petersson and R. H. Mach, *Chemical Communications*, 2020, **56**, 3567.
8. Y. Xiao, B. Ma, D. Mcelheny, S. Parthasarathy, F. Long, M. Hoshi, R. Nussinov and Y. Ishii, *Nat. Struct. Mol. Biol.*, 2015, **22**, 499.
9. B. J. Kuipers and H. Gruppen, *J. Agric. Food Chem.*, 2007, **55**, 5445.
10. C. M. Haney, C. L. Cleveland, R. F. Wissner, L. Owei, J. Robustelli, M. J. Daniels, M. Canyurt, P. Rodriguez, H. Ischiropoulos, T. Baumgart and E. J. Petersson, *Biochemistry*, 2017, **56**, 683.
11. M. J. Daniels, J. B. Nourse, H. Kim, V. Sainati, M. Schiavina, M. G. Murrali, B. Pan, J. J. Ferrie, C. M. Haney, R. Moons, N. S. Gould, A. Natalello, R. Grandori, F. Sobott, E. J. Petersson, E. Rhoades, R. Pierattelli, I. Felli, V. N. Uversky, K. A. Caldwell, G. A. Caldwell, E. S. Krol and H. Ischiropoulos, *Sci. Rep.*, 2019, **9**, 2937.
12. R. Kiss, M. Sandor and F. A. Szalai, *J. Cheminformatics*, 2012, **4**, P17.
13. R. J. Karpowicz, C. M. Haney, T. S. Mihaila, R. M. Sandler, E. J. Petersson and V. M. Y. Lee, *J. Biol. Chem.*, 2017, **292**, 13482.
14. K. C. Luk, V. Kehm, J. Carroll, B. Zhang, P. O'brien, J. Q. Trojanowski and V. M. Y. Lee, *Science*, 2012, **338**, 949.
15. C. Peng, R. J. Gathagan, D. J. Covell, C. Medellin, A. Stieber, J. L. Robinson, B. Zhang, R. M. Pitkin, M. F. Olufemi, K. C. Luk, J. Q. Trojanowski and V. M. Y. Lee, *Nature*, 2018, **557**, 558.
16. G. M. Morris, R. Huey, W. Lindstrom, M. F. Sanner, R. K. Belew, D. S. Goodsell and A. J. Olson, *J. Comput. Chem.*, 2009, **30**, 2785.
17. B. S. Li, P. Ge, K. A. Murray, P. Sheth, M. Zhang, G. Nair, M. R. Sawaya, W. S. Shin, D. R. Boyer, S. L. Ye, D. S. Eisenberg, Z. H. Zhou and L. Jiang, *Nat. Commun.*, 2018, **9**, 10.
18. Y. Li, C. Zhao, F. Luo, Z. Liu, X. Gui, Z. Luo, X. Zhang, D. Li, C. Liu and X. Li, *Cell Res.*, 2018, **28**, 897.
19. M. Schweighauser, Y. Shi, A. Tarutani, F. Kametani, A. G. Murzin, B. Ghetti, T. Matsubara, T. Tomita, T. Ando, K. Hasegawa, S. Murayama, M. Yoshida, M. Hasegawa, S. H. W. Scheres and M. Goedert, *bioRxiv*, 2020, DOI: 10.1101/2020.02.05.935619, 2020.02.05.935619.
20. Silicos-It, Silicos-It Chemoinformatics Services and Software, http://silicos-it.be.s3-website-eu-west-1.amazonaws.com/index.html).

21. Beta_Nov16, beta nov16, https://www.rosettacommons.org/docs/latest/Updates-beta-nov16).
22. H. Park, P. Bradley, P. Greisen, Y. Liu, V. K. Mulligan, D. E. Kim, D. Baker and F. Dimaio, *J Chem Theory Comput*, 2016, **12**, 6201.
